# Long-term outcomes and response to treatment in diacylglycerol kinase epsilon nephropathy

**DOI:** 10.1016/j.kint.2020.01.045

**Published:** 2020-06

**Authors:** Vicky Brocklebank, Gurinder Kumar, Alexander J. Howie, Jayanthi Chandar, David V. Milford, Janet Craze, Jonathan Evans, Eric Finlay, Michael Freundlich, Daniel P. Gale, Carol Inward, Martin Mraz, Caroline Jones, William Wong, Stephen D. Marks, John Connolly, Bronte M. Corner, Kate Smith-Jackson, Patrick R. Walsh, Kevin J. Marchbank, Claire L. Harris, Valerie Wilson, Edwin K.S. Wong, Michal Malina, Sally Johnson, Neil S. Sheerin, David Kavanagh

**Affiliations:** 1 National Renal Complement Therapeutics Centre, Newcastle upon Tyne Hospitals NHS Foundation Trust, Newcastle upon Tyne, UK; 2 Complement Therapeutics Research Group, Translational and Clinical Research Institute, Newcastle University, Newcastle upon Tyne, UK; 3 Division of Paediatric Nephrology, Sheikh Khalifa Medical City, Abu Dhabi, United Arab Emirates; 4 Birmingham Women’s and Children’s NHS Foundation Trust, Birmingham, UK; 5 Division of Pediatric Nephrology, University of Miami, Miami, Florida, USA; 6 Department of General Paediatrics, Oxford University Hospitals NHS Foundation Trust, Oxford, UK; 7 Children’s Renal and Urology Unit, Nottingham Children’s Hospital, Nottingham University Hospitals NHS Foundation Trust, Nottingham, UK; 8 Leeds Teaching Hospitals NHS Trust, Leeds, UK; 9 Department of Renal Medicine, University College London, UK; 10 Department of Paediatric Nephrology, Bristol Royal Hospital For Children, University Hospitals Bristol NHS Foundation Trust, Bristol, UK; 11 Department of Paediatric Nephrology, Alder Hey Children’s Hospital NHS Trust, Liverpool, UK; 12 Department of Paediatric Nephrology, Starship Children’s Hospital, Grafton, Auckland, New Zealand; 13 Department of Paediatric Nephrology, Great Ormond Street Hospital for Children NHS Foundation Trust, London, UK; 14 Centre for Nephrology, Royal Free Hospital, University College London, London, UK; 15 Great North Children’s Hospital, Sir James Spence Institute, Royal Victoria Infirmary, Newcastle, UK

**Keywords:** atypical hemolytic uremic syndrome, diacylglycerol kinase ε, membranoproliferative glomerulonephritis, thrombotic microangiopathy

## Abstract

Recessive mutations in diacylglycerol kinase epsilon (*DGKE*) display genetic pleiotropy, with pathological features reported as either thrombotic microangiopathy or membranoproliferative glomerulonephritis (MPGN), and clinical features of atypical hemolytic uremic syndrome (aHUS), nephrotic syndrome or both. Pathophysiological mechanisms and optimal management strategies have not yet been defined. In prospective and retrospective studies of aHUS referred to the United Kingdom National aHUS service and prospective studies of MPGN referred to the National Registry of Rare Kidney Diseases for MPGN we defined the incidence of DGKE aHUS as 0.009/million/year and so-called DGKE MPGN as 0.006/million/year, giving a combined incidence of 0.015/million/year. Here, we describe a cohort of sixteen individuals with DGKE nephropathy. One presented with isolated nephrotic syndrome. Analysis of pathological features reveals that *DGKE* mutations give an MPGN-like appearance to different extents, with but more often without changes in arterioles or arteries. In 15 patients presenting with aHUS, ten had concurrent substantial proteinuria. Identified triggering events were rare but coexistent developmental disorders were seen in six. Nine with aHUS experienced at least one relapse, although in only one did a relapse of aHUS occur after age five years. Persistent proteinuria was seen in the majority of cases. Only two individuals have reached end stage renal disease, 20 years after the initial presentation, and in one, renal transplantation was successfully undertaken without relapse. Six individuals received eculizumab. Relapses on treatment occurred in one individual. In four individuals eculizumab was withdrawn, with one spontaneously resolving aHUS relapse occurring. Thus we suggest that DGKE-mediated aHUS is eculizumab non-responsive and that in individuals who currently receive eculizumab therapy it can be safely withdrawn. This has important patient safety and economic implications.

## Graphical abstract

**Figure:**
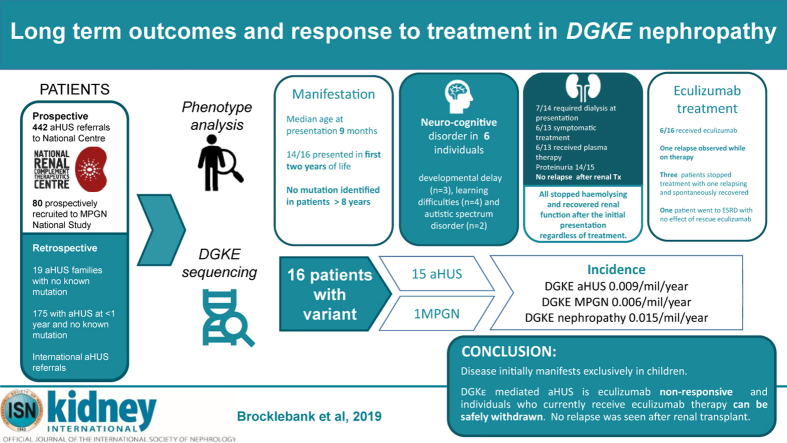

In 2013 recessive mutations in *DGKE*, which encodes diacylglycerol kinase epsilon (DGKE), were first reported to cause atypical hemolytic uremic syndrome (aHUS)^1^ and nephrotic syndrome, with glomerular microangiopathy said to resemble membranoproliferative (mesangiocapillary) glomerulonephritis (MPGN)^2^ (Online Mendelian Inheritance in Man #615008), though the pathophysiological mechanisms remain poorly understood.

aHUS is characterized by a clinical presentation with thrombocytopenia, microangiopathic hemolytic anemia, and organ injury.^3^ aHUS is a broad term that has been used to refer to cases of thrombotic microangiopathy (TMA), in which thrombotic thrombocytopenic purpura and shiga toxin–producing *E*sc*herichia coli* HUS have been excluded.^3^ Most individuals with aHUS in whom all secondary causes of TMA have been excluded^3^ have complement-mediated aHUS—in ∼50% an inherited or acquired complement abnormality is identified,^4^ and others will improve concurrent with complement-inhibiting therapy despite no complement abnormality being detected.

MPGN-type lesions, which can manifest in a diverse range of conditions, including aHUS, are characterized by a histopathological pattern recognized by light microscopy, comprising an increase in mesangial cellularity and matrix with thickening of glomerular capillary walls secondary to subendothelial deposition of immune complexes and/or complement factors, cellular entrapment, and new basement membrane formation. Subclassification was historically based on electron microscopy (EM) appearances.^5^ The current classification system of MPGN includes subendothelial-type MPGN, dense deposit disease, and C3 glomerulonephritis and considers the underlying pathogenesis and therefore informs the approach to investigation and management.^6^^,^^7^ Ozaltin *et al.* described glomerular microangiopathy with histological features of both endothelial distress and MPGN, with peripheral segmental deposition of IgM and no intraglomerular deposition of C3, in 8 individuals with *DGKE* mutations.^2^ When pathological features are analyzed, it was found that the initial articles on *DGKE* mutations^1^^,^^2^ probably describe the same glomerular appearances albeit with different phenotypic presentations.

DGKE plays an important role in cellular signaling by converting diacylglycerol to phosphatidic acid in the phosphatidylinositol cycle.^8^ Diacylglycerol activates protein kinase C, which leads to a number of downstream effects including changes in vascular tone,^9^^,^^10^ the release of prothrombotic factors^1^^,^^11^ and antithrombotic factors as well as platelet activation,^1^ and changes in the actin cytoskeleton.^12^ DGKE phosphorylates diacylglycerol and therefore regulates this signaling pathway.^8^ The mechanisms by which DGKE abnormalities result in TMA/MPGN have not been defined. *DGKE* is expressed in podocytes and affects intracellular diacylglycerol concentration.^2^ *DGKE* knockout mice have no apparent renal phenotype, but have subclinical abnormalities of the glomerular endothelium and basement membrane on EM, develop glomerular capillary occlusion when exposed to nephrotoxic serum, and have impaired production of cyclooxygenase 2 and prostaglandin E_2_.^13^

Currently, DGKE nephropathy manifesting in aHUS has been reported in 35 individuals^1^^,^^14^, ^15^, ^16^, ^17^, ^18^, ^19^ and a nephrotic syndrome/MPGN-like phenotype in 9 individuals.^2^ The collective characteristics were summarized recently by Azukaitis *et al.*,^19^ although without a detailed investigation of reported pathological diagnoses. The role of complement activation in DGKE nephropathy has not been definitively established. Markers suggestive of complement activation have been observed in some patients, but relapses have occurred despite complement inhibiting therapy in 2 individuals.^19^ Concurrent *DGKE* and *C3* mutations have been reported,^15^ but the consequences of *DGKE* loss of function appear to occur independently of complement *in vitro*.^20^

Here we determine the incidence of DGKE aHUS and MPGN in prospective studies in the United Kingdom, review the prevalence in historic aHUS cohorts, describe the clinical presentation and long-term outcomes, and report the response to treatment and transplantation.

## Results

### Incidence of DGKE-mediated aHUS and MPGN

Sixteen individuals (5 boys, 11 girls) with DGKE nephropathy were identified by systematic genetic analysis of 5 cohorts referred to the National Renal Complement Therapeutics Centre (NRCTC, http://www.atypicalhus.co.uk/), Newcastle upon Tyne, UK.

Prospective screening of all UK incident patients with aHUS was undertaken at the national specialized aHUS service commissioned by NHS England in April 2013.^21^ All individuals in England with a suspected diagnosis of aHUS are referred to the NRCTC. In the 6 years (2013–2019) since commissioning, 442 individuals have been referred with a tentative diagnosis of aHUS and 3 individuals (NCL34, NCL39, and NCL40) were identified in the prospective cohort. The incidence rate of complement-mediated aHUS is 0.47 per million per year, and the incidence rate of DGKE aHUS is 0.009 per million per year.

A prospective study of children in the United Kingdom referred to the UK MPGN pediatric RaDaR (The UK National Registry of Rare Kidney Diseases) with MPGN, dense deposit disease, or C3 glomerulonephritis was conducted from 2012 to 2015. During this period, 80 children were screened and 1 patient (NCL37) was identified. The incidence rate of so-called DGKE MPGN is ∼0.006 per million per year.

A retrospective analysis of 19 families referred to the NRCTC in whom no known genetic or autoimmune cause of aHUS had been identified was undertaken using whole exome sequencing. This identified 3 families (4 individuals): NCL25, NCL26, NCL27, and NCL29. An additional retrospective analysis of prevalent patients referred to the NRCTC was undertaken using Sanger sequencing. Of 175 UK individuals who presented with aHUS before the age of 18 years, 49 had no complement genetic or autoimmune cause identified and were analyzed. This identified 4 individuals: NCL28, NCL30, NCL33, and NCL36.

The NRCTC receives international referrals for genetic screening of patients, both incident and prevalent, presenting with aHUS and MPGN. This identified 4 patients: NCL31, NCL32, NCL35 (United Arab Emirates), and NCL38 (New Zealand).

### Clinical presentation

**Figure 1:**
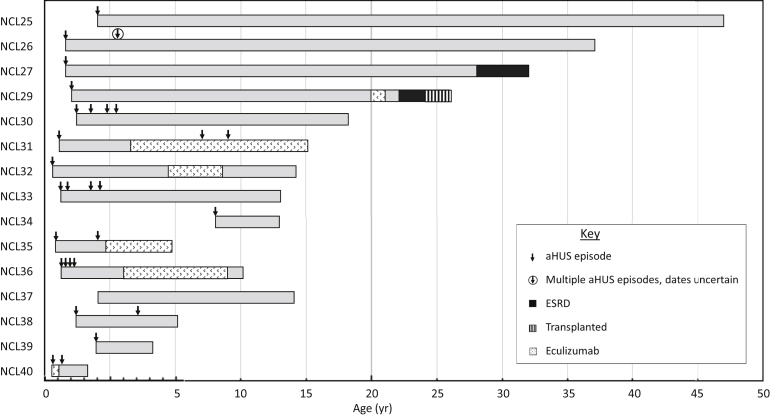
**Age at presentation and clinical course.** All but 1 individual presented in the first 2 years of life, and most individuals experienced thrombotic microangiopathy relapses in early childhood. Two individuals have developed end-stage renal disease (ESRD) >20 years after the initial presentation, and 1 has received a kidney transplant. Six individuals have been treated with eculizumab, 1 with relapses on treatment, and in 4, it has been withdrawn. NCL28 is not included in the figure because of insufficient data being available. The scale is increased for the first 5 years of life to depict the detail of the relapses. aHUS, atypical hemolytic uremic syndrome.

Detailed data on clinical presentation were available for 15 individuals. The median age at presentation was 9 months, with 14 of 15 presenting in the first 2 years of life (see Figure 1).The mean follow-up period was 19 years (range, 1–45 years). Clinical and laboratory features at the initial presentation are summarized in Tables 1 and 2. Prodromal diarrhea was observed in 4 of 14 patients, and infection was reported as a triggering event in 3 of 14. Six individuals have been diagnosed with ≥1 developmental disorder: developmental delay (*n* = 3), learning difficulties (*n* = 4), and autistic spectrum disorder (*n* = 2).

**Table 1:** Clinical and laboratory features at the initial presentation with DGKE nephropathy

Clinical feature	Number of patients
Age (mo) (*n* = 15)	Mean 17; median 9; range 3–96
Sex (*n* = 16)	
Male	5
Female	11
Prodrome (*n* = 14)	
Vomiting	2
Diarrhea	4
Triggering event (*n* = 14)	
None identified	11
Infection	3
Clinical features (n = 14)	
Proteinuria	14
Nephrotic range proteinuria	10
Hematuria	14
Hypertension^a^	12
Extrarenal manifestations (*n* = 13)	
None	9
Neurological	4
Medical history (*n* = 14)	
Developmental delay	3
Learning difficulties	4
Autistic spectrum disorder	2

Laboratory feature	Mean	Range
Hemoglobin level (g/l) (*n* = 13)	76	49–111
Platelet count (×10^9^) (*n* = 13)	120	15–315
Creatinine level (μmol/l) (*n* = 13) Albumin level (g/l) (*n* = 11)	311 23	54–1921 15–32
Lactate dehydrogenase level (U/l) (*n* = 10)	2651	652–5909
C3 level (0.68–1.38) (g/l) (*n* = 14)	1.04	0.50–1.64
C4 level (0.18–0.6) (g/l) (*n* = 13)	0.28	0.07–0.65
Urine ACR (if quantified) (mg/mmol) (*n* = 8)	1069	200–3447

ACR, albumin/creatinine ratio; DGKE, diacylglycerol kinase epsilon.

a Hypertension was defined according to the international guidelines.

**Table 2:** Demographic, genetic, and laboratory characteristics at presentation

Patient	Sex	Age at diagnosis	Clinical presentation	Renal biopsy	C3 level (0.68–1.38) (g/l)	C4 level (0.18–0.6) (g/l)	*DGKE* mutation	Inheritance	Consanguinity	Affected siblings^a^	Complement genetic analysis	Anti-FH Ab
NCL25	F	2 yr	aHUS	Yes	0.95^b^	0.15^b^	c.463A>G p.(Arg155Gly)^c^ c.1427T>C p.(Leu476Pro)^c^	Comp Het	No	Yes	NMD	No
NCL26	F	1 yr 10 mo	aHUS	Yes	NA	NA	c.826delG p.(Val276Phefs∗8)^c^	Hom	Yes	Yes	NMD	No
NCL27^d^	M	9 mo	aHUS/NS	Yes	0.5	0.12	c.1597A>C p.(Thr533Pro)^c^	Hom	No	Yes	NMD	No
NCL28	F	NA	aHUS	ND	1.01	0.22	c.1A>T p.(Met1Leu)^c^	Hom	Yes	NA	NMD	No
NCL29^d^	M	12 mo	aHUS/NS	Yes	0.52	NA	c.1597A>C p.(Thr533Pro)^c^	Hom	No	Yes	NMD	No
NCL30	F	2 yr 2 mo	aHUS	ND	0.89	0.07	c.393C>G p.(Asn131Lys)^c^ c.465-2A>G^c^	Comp Het	No	No	NMD	No
NCL31	F	8 mo	aHUS/NS	ND	1.06	0.28	c.325A>G p.(Lys109Glu)	Hom	Yes	No	NMD	No
NCL32	F	3 mo	aHUS/NS	ND	0.94	0.18	c.236A>C p.(Gln79Pro)^c^	Hom	Yes	No	NMD	ND
NCL33	F	7 mo	aHUS	ND	1.64	0.65	c.966G>A p.(Trp322∗)	Hom	No	No	NMD	No
NCL34	M	8 yr	aHUS	Yes	1.15	0.34	c.1647_1650delAACA p.(Thr550Metfs∗13)^c^	Hom	Yes	No	NMD	No
NCL35	F	6 mo	aHUS/NS	ND	1.44	0.28	c.325A>G p.(Lys109Glu)	Hom	Yes	No	NMD	ND
NCL36	M	7 mo	aHUS	ND	0.55	0.18	c.966G>A p.(Trp322∗) c.1524+2T>C	Comp Het	No	No	NMD	ND
NCL37	F	2 yr 1 mo	NS	Yes	1.13	0.24	c.323G>A p.(Cys108Tyr)^c^	Hom	Yes	No	NMD	No
NCL38	M	1 yr 2 mo	aHUS	ND	0.9	0.16	c.966G>A p.(Trp322∗)	Hom	Yes	No	NMD	No
NCL39	F	1 yr 11 mo	aHUS	ND	1.44	0.44	c.966G>A p.(Trp322∗) c.465-2A>G^c^	Comp Het	No	No	NMD	No
NCL40	F	3 mo	aHUS	ND	1.37	0.24	c.966G>A p.(Trp322∗)	Hom	No	No	NMD	No

aHUS, atypical hemolytic uremic syndrome; anti-FH Ab, anti–factor H autoantibody; Comp Het, compound heterozygote; DGKE, diacylglycerol kinase epsilon; F, female; Hom, homozygote; M, male; NA, not available; ND, not done; NMD, no mutation detected; NS, nephrotic syndrome.

Normal ranges for C3 and C4 are shown in parentheses.

a See Supplementary Figure S2 for pedigrees.

b Convalescent sample.

c Not previously reported.

d Siblings.

Fifteen of 16 patients presented with a clinical diagnosis of aHUS; 8 of 15 had concurrent nephrotic range proteinuria, and a further 3 of 15 who did not have proteinuria quantified at presentation had low serum albumin levels. One patient (NCL37) presented with nephrotic syndrome and no features of TMA. The serum creatinine and proteinuria values are presented in Table 3 and Supplementary Figure S1.

**Table 3:** Presentation at the initial episode, management, and evolution

Patient	Creatinine level (μmol/l)	Proteinuria urine ACR (mg/mmol)	Serum albumin level (g/l)	MAHA	Management						Complications and outcome
					NA	Supportive	Dialysis (duration)	FFP	PEX	Eculizumab	
NCL25	NA	NA	NA	NA	Yes	NA	No	NA	NA	No	NA
NCL26	NA	NA	NA	Yes	Yes	NA	NA	NA	NA	No	Spontaneous remission
NCL27	88	(1.58 g/24 h)	NA	NA	–	Yes	No	No	No	No	Recovery of renal function
NCL28	NA	NA	NA	NA	Yes	NA	NA	NA	NA	No	NA
NCL29	141	2660	NA	NA	–	Yes	Yes (17 d)	Yes (×7)	No	No	Recovery of renal function
NCL30	150	1268	23	Yes	–	Yes	No	No	No	No	Recovery of renal function
NCL31	165	>500	18	Yes	–	Yes	No	Yes	No	No	NA
NCL32	312	>500	15	Yes	–	Yes	Yes (9 mo)	Yes	No	No	NA
NCL33	210	NA	26	Yes	–	Yes	Yes (17 d)	No	No	No	Recovery of renal function
NCL34	1921	PCR 339a	32	Yes	–	Yes	Yes (12 d)	No	No	No	Recovery of renal function
NCL35	112	>500	23	Yes	–	Yes	No	Yes	No	No	NA
NCL36	68	PCR 2410b	25	Yes	–	Yes	Yes (12 d)	No	Yes	No	Partial recovery of renal function
NCL37	54	309	24	No	–	Yes steroid + ACEI	No	No	No	No	Remission
NCL38	145	3447	27	Yes	–	Yes	Yes (15 d)	Yes	Yes	No	Recovery Hypertensive heart failure and encephalopathy
NCL39	453	1836	17	Yes	–	Yes	Yes (15 d)	No	No	No	Spontaneous remission
NCL40	223	PCR 3857b	28	Yes	–	Yes	No	No	No	Yes	Remission

ACEI, angiotensin-converting enzyme inhibitor; ACR, albumin/creatinine ratio; FFP, fresh frozen plasma; MAHA, microangiopathic hemolytic anemia (anemia and schistocytes identified on blood film microscopy); NA, not available; PCR, protein/creatinine ratio; PEX, plasma exchange.

If proteinuria was not quantified at presentation, the earliest available sample is shown: ^a^1 mo and ^b^3 mo after presentation.

### Pathological findings

**Figure 2:**
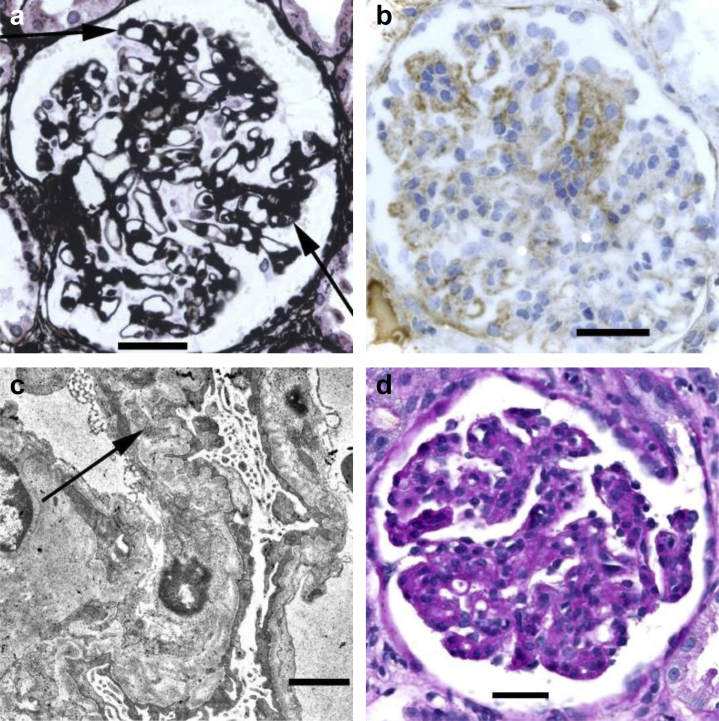
**Pathological appearances.** (**a–c**) Glomeruli in the renal biopsy in NCL34. (**a**) Periodic acid–methenamine silver staining shows mild segmental mesangial expansion, slight doubling of basement membranes (arrows), and an adhesion to the Bowman’s capsule at the tubular origin. Bar = 50 μm. (**b**) Immunoperoxidase staining shows a patchy subendothelial deposition of IgG. Bar = 50 μm. (**c**) Electron microscopy shows irregular wrinkling of glomerular basement membranes, patchy doubling of basement membranes with subendothelial widening and mesangial interposition (arrow), and effacement of podocyte foot processes. Bar = 1 μm. (**d**) Glomerulus in the renal biopsy in NCL37. Periodic acid–Schiff staining shows global mesangial expansion and hypercellularity, with a widespread doubling of basement membranes. Bar = 50 μm. To optimize viewing of this image, please see the online version of this article at www.kidney-international.org.

Only 1 renal biopsy was available for detailed microscopic review (NCL34, who presented with an aHUS phenotype). This showed segmental mesangial hypercellularity and slight doubling of glomerular basement membranes (GBMs) in a few glomeruli, most of which were normal (Figure 2a). Deposition of immunoproteins was seen only in the segmental lesions (Figure 2b), and there was a loose subendothelial material in these areas on EM (Figure 2c). Arterioles and arteries were normal. Images and a report were available on another biopsy (NCL37, who presented with isolated nephrotic syndrome). This was reported to show global and diffuse doubling of basement membranes, with segmental endocapillary hypercellularity, mesangial and subendothelial deposition of IgM and a little IgG but no C3, and mesangial and subendothelial deposits on EM (Figure 2d). Blood vessels were normal. Histology reports were available on 3 biopsies (NCL25, NCL27, and NCL29). NCL25 was reported to show doubling of many basement membranes with segmental deposition of IgM and a little C3, with severe hyalinosis and narrowing of arterioles. NCL27 showed doubling of some basement membranes with mesangial and subendothelial deposition of IgM and a little IgG and IgA, with corresponding deposits on EM and normal blood vessels. NCL29 showed segmental doubling of basement membranes with predominantly subendothelial deposition of IgM and C3, and normal blood vessels. The only information available about NCL26 was that there was loose concentric intimal thickening in a small artery.

### Complement analysis

Of 12 patients with serum available, 3 had low C3 levels and 4 had low serum C4 levels (Table 2; Supplementary Figure S1). In 5 of 5 individuals, factor H levels were normal, and in 2 of 5 individuals, factor I levels were low. Factor H autoantibodies were not detected in any individual.

### Genetic analysis

The results of the genetic analysis are presented in Table 2. Twelve of 16 individuals had homozygous mutations (consanguinity reported in 8), and 4 of 16 individuals had compound heterozygous mutations in *DGKE.* The cohort includes 2 siblings (NCL27 and NCL29). Two other individuals (NCL25 and NCL26) had siblings with HUS who died >30 years ago who are not included in our cohort because no genetic analysis was possible, and 1 individual (NCL34) has 3 siblings who have the same homozygous mutation but are asymptomatic; pedigrees for these 4 families are shown in Supplementary Figure S2.

**Figure 3:**
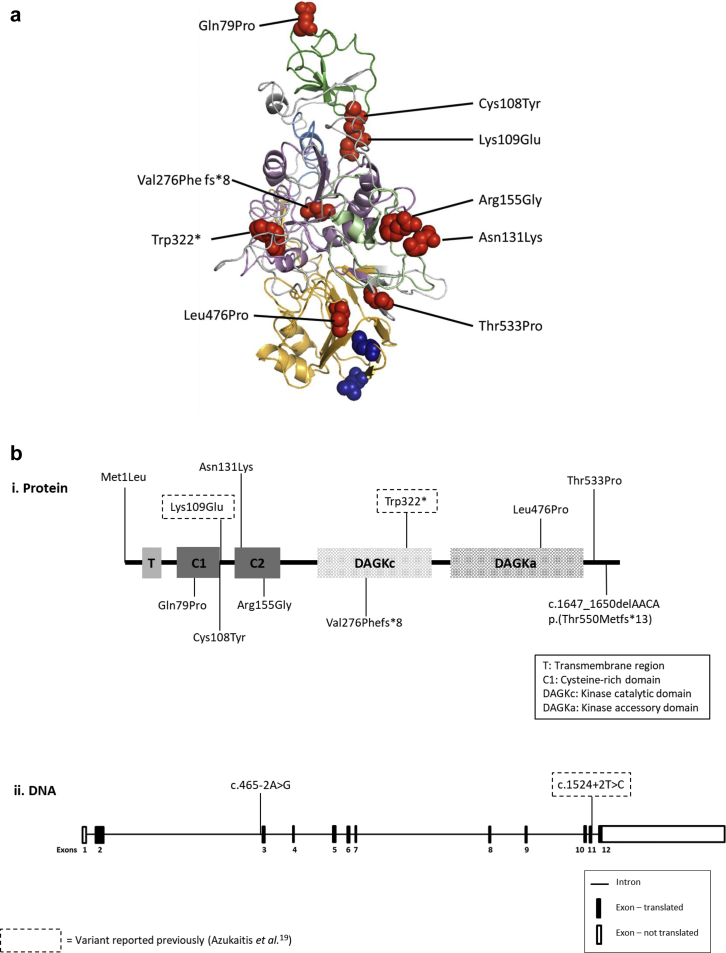
***DGKE* mutations.** (**a**) Predicted protein model of diacylglycerol kinase epsilon (DGKE) demonstrating positions of rare genetic variants. DGKE protein model demonstrating positions of rare genetic variants. Phyre2 was used to create a predicted protein model of DGKE. Amino acid substitutions are shown by red spheres. Transmembrane domain (amino acids 22–42) is shown in blue, cysteine-rich domain 1 (amino acids 60–109) in dark green, cysteine-rich domain 2 (amino acids 125–178) in light green, kinase catalytic domain (amino acids 219–350) in pink, and kinase accessory domain (amino acids 369–524) in orange. Amino acids L431 and L438, thought to be important for diacylglycerol specificity, are shown in blue spheres. (**b**) Schematic representation of (i) DGKE protein and (ii) *DGKE* DNA sequence showing relative positions of variants.

**Figure 4:**
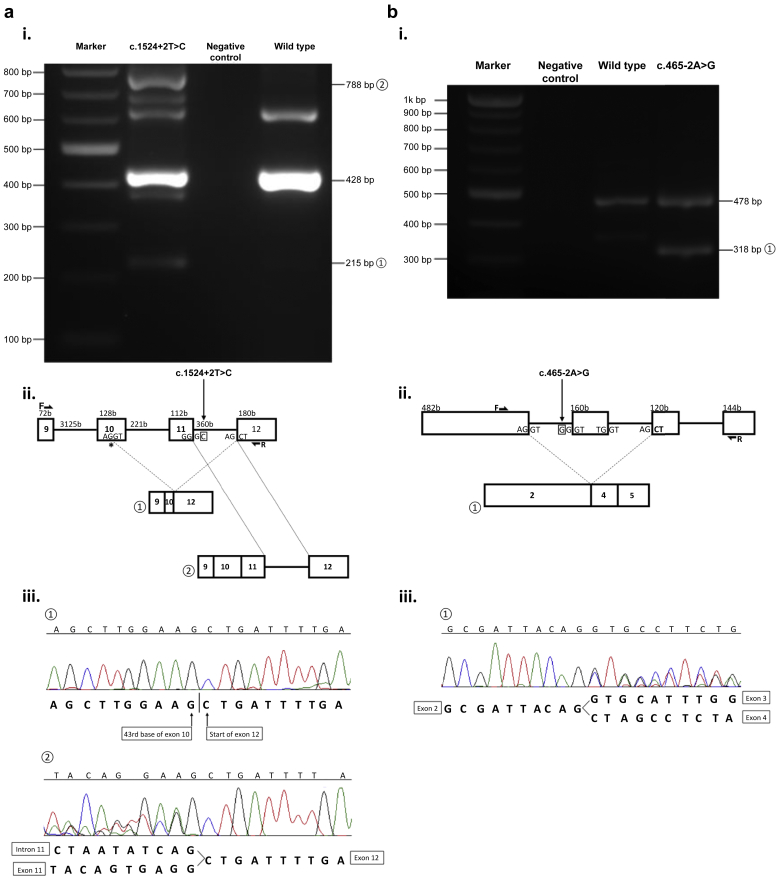
***DGKE* RNA studies.** PCR, gel electrophoresis, and sequence analysis of cDNA reverse transcribed from peripheral blood lymphocytes were performed and demonstrated abnormal splicing for both c.1524+2T>C and c.465-2A>G. (**a**) NCL36: c.1524+2T>C. Three transcripts were detected on sequencing and are labeled on the (i) gel and (ii) diagram: wild type (428 bp); splice variant ① (215 bp): this corresponds to the first 43 bases of exon 10 spliced to exon 12 because of a cryptic splice site (∗) in exon 10; splice variant ② (788 bp): this represents a gain of 360 bp that corresponds to the inclusion of the whole of intron 11. The Sanger sequencing traces are shown in (iii). The other PCR products visible on the gel were of insufficient intensity to be adequately sequenced. (**b**) NCL30 and NCL39: c.465-2A>G. Two transcripts were detected and shown on the (i) gel and (ii) diagram: wild type (478 bp) and an abnormal transcript ①; at 318 bp, this represents a deletion of 160 bp that corresponds to the whole of exon 3. The Sanger sequencing trace is shown in (iii). The positions of forward (F) and reverse (R) primers are shown. Details of the primer design are available in Supplementary Figure S6.

Rare genetic variants (Supplementary Figure S3) were identified throughout the DGKE molecule (Figure 3). All *DGKE* variants we identified are predicted to be functionally significant on the basis of conservation (Supplementary Figure S4) and *in silico* analysis (Supplementary Table S1). RNA studies of the predicted splice site mutations identified in NCL36 (c.1524+2T>C) and NCL30 and NCL39 (c.465-2A>G) confirmed the presence of abnormally spliced products (Figure 4). No coexistent mutations in *CFH*, *CFI*, *CD46*, *C3*, *CFB*, and *MMACHC* were identified.

### Evolution of the initial presentation

The treatment and evolution of the initial presentation are summarized in Table 3. Information on management was available for 14 of 16 patients. Half of the patients (7 of 14) required dialysis at presentation; in 6 of 7 the duration was <3 weeks, and in 1 of 7 the duration was 9 months. Of 13 of 15 presenting with aHUS, 6 of 13 received supportive management, 6 of 13 received plasma therapy (fresh frozen plasma or plasma exchange), and 1 received eculizumab at the time of the initial presentation. All individuals discontinued hemolyzing and recovered renal function after the initial presentation regardless of the treatment. The single individual (NCL37) who presented with only nephrotic syndrome was treated with angiotensin-converting enzyme inhibition and corticosteroids, and remission was achieved.

### Progression and long-term outcomes

Progression and long-term outcomes are summarized in Figure 1 and Table 4. Of 14 individuals who presented with aHUS in whom detailed clinical data were available, 9 had TMA relapses. In 6 of 9, multiple relapses occurred. In 8 of 9, all relapses occurred before the age of 5 years (in the other individual, the final relapse occurred at the age of 8 years).

**Table 4:** Management and long-term outcomes

Patient	Age now	Duration of FU	Maintenance treatment	TMA relapses	Management of relapses	Clinical characteristics at last FU				ESRD (age)	Transplanted (age)
						eGFR (ml/min per 1.73 m^2^) (CKD stage)	Proteinuria urine ACR or PCR (mg/mmol)	Hematuria (dipstick positive)	Hypertension (number of drugs)		
NCL25	47 yr	45 yr	None	NA	na	63 (CKD G2)	NA	NA	NA	No	No
NCL26	37 yr	35 yr	None	Multiple	PEX	30 (CKD G3b)	NA	NA	Yes (2)	No	No
NCL27	32 yr	31 yr	None	No	na	ESRD	NA	Yes	Yes	Yes (28 yr)	No
NCL28	27 yr	Lost to FU	None	Multiple	NA	NA	NA	NA	NA	NA	NA
NCL29	26 yr	25 yr	Eculizumab 2013–2014	No	na	Transplant: creatinine level 106 μmol/l	PCR 0.06	NA	NA	Yes (22 yr)	Yes (24 yr)
NCL30	18 yr	16 yr	None	3	Supportive	79 (CKD G2 A3)	ACR 275	2+	Yes (1)	No	No
NCL31	15 yr 6 mo	15 yr	Eculizumab	2, while on eculizumab	FFP, PEX, eculizumab	42 (CKD G3b A3)	ACR 262	1+	Yes (1)	No	No
NCL32	14 yr	14 yr	Eculizumab 2013–2016	0	na	34 (CKD G3b A3)	ACR 182	1+	Yes (2)	No	No
NCL33	13 yr 8 mo	13 yr	None	3	Supportive, FFP (1 yr), PEX	42 (CKD G3b A3)	ACR 409	3+	Yes (3)	No	No
NCL34	13 yr 7 mo	5 yr 7 mo	None	0	na	88 (CKD G1 A2)	ACR 6.4	No	No	No	No
NCL35	11 yr 2 mo	11 yr	Eculizumab	1, before eculizumab	FFP, PEX, eculizumab	174 (CKD G1 A3)	ACR 378	1+	Yes (2)	No	No
NCL36	10 yr 6 mo	10 yr	PEX 2009–2012 Eculizumab 2012–2018	3, before eculizumab	PEX	103 (CKD G1 A3)	PCR 186	Yes	Yes (2)	No	No
NCL37	14 yr 8 mo	12 yr	None	na No NS relapse	na	90 (CKD G1 A3)	PCR 67	Yes	Yes (1)	No	No
NCL38	5 yr 7 mo	4 yr 5 mo	None	1	Supportive, FFP, PEX	Creatinine level 23 μmol/l (CKD G1 A3)	PCR 284	No	Yes (4)	No	No
NCL39	4 yr 7 mo	2 yr 8 mo	None	0	na	74 (CKD G2 A3)	ACR 74	1+	Yes (1)	No	No
NCL40	2 yr	1 yr 9 mo	Eculizumab 2018 for 3 mo	1, 6 wk after discontinuing eculizumab	Supportive	>90 (CKD G1 A1)	ACR <50	NA	No	No	No

ACR, albumin/creatinine ratio; CKD, chronic kidney disease; eGFR, estimated glomerular filtration rate; ESRD, end-stage renal disease; FFP, fresh frozen plasma; FU, follow-up; NA, not available; na, not applicable; NS, nephrotic syndrome; PCR, protein/creatinine ratio; PEX, plasma exchange; TMA, thrombotic microangiopathy.

The eGFR was calculated as follows: for children (<18 yr), the Schwartz formula was used: eGFR (ml/min per 1.73 m^2^) = (0.55 × height [cm] × *K* [constant])/serum creatinine level (μmol/l) × 0.0113 (correction factor for mg/dl); in the first year of life, for preterm babies, *K* = 0.33 and for full-term infants, *K* = 0.45; for infants and children between ages of 1 and 12 yr, *K* = 0.55; and for adolescent boys, *K* = 0.7. For adults, the Chronic Kidney Disease Epidemiology Collaboration equation was used: 141 × min(*S*_cr_/*κ*, 1)^*α*^ × max(*S*_cr_/*κ*, 1)^−1.209^ × 0.993^Age^ × 1.018 [if female] × 1.159 [if black], where *S*_cr_ is serum creatinine level in mg/dl, *κ* is 0.7 for females and 0.9 for males, *α* is −0.329 for females and −0.411 for males, min indicates the minimum of *S*_cr_/*κ* or 1, and max indicates the maximum of *S*_cr_/*κ* or 1.

One individual (NCL36) received maintenance plasma exchange therapy for 3 years and experienced multiple relapses, thought to have been triggered by viral infections, while on treatment. Six individuals received eculizumab therapy (Figure 1). In the 2 UK residents, eculizumab was withdrawn immediately after the identification of mutations in *DGKE* and 16 months later 1 has had no relapses and the other individual had a relapse 6 weeks after withdrawal and spontaneously remitted with supportive management.

In non-UK residents, eculizumab was withdrawn in 1 patient (NCL32) after 3 years, with no relapses 3 years on, and another patient with progressive chronic kidney disease (CKD) and now end-stage renal disease (ESRD) (NCL29) was treated with eculizumab for 12 months with no appreciable clinical response. Two non-UK residents (NCL31 and NCL35) continue on maintenance eculizumab; 1 has experienced relapses while on treatment.

**Figure 5:**
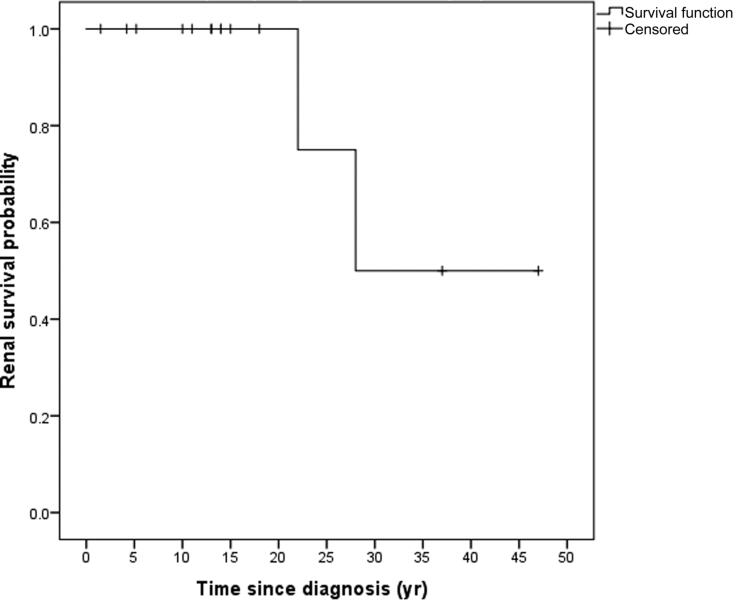
**Kaplan-Meier curve for renal survival.** Two individuals reached end-stage renal disease >20 years after the initial presentation.

Long-term outcome data are available for 15 of 16 individuals. Two individuals, siblings homozygous for c.1597A>C p.(Thr533Pro), who initially presented with clinical features of aHUS, have developed ESRD >20 years after the initial presentation. Figure 5 shows the Kaplan-Meier curve for renal survival. A Kaplan-Meier curve comparing individuals who received eculizumab with those who did not is included in Supplementary Figure S5. Relatives of individuals in our cohort in whom genetic analysis was not possible were not included in the survival analysis. We recognize that this introduces bias and have included a Kaplan-Meier curve showing renal and patient survival that does include these individuals in Supplementary Figure S5. All individuals who have not developed ESRD have CKD: glomerular filtration rate stage G1 (*n* = 6), stage G2 (*n* = 3), and stage G3 (*n* = 4). Data on proteinuria are available for 11 of 16 individuals, quantified as albuminuria category A1 in 1 of 11, A2 in 1 of 11, and A3 in 9 of 11. Nine of 11 have documented persistent nonvisible hematuria. Blood pressure measurement data are available for 13 individuals. Eleven of 13 have hypertension or are taking antihypertensive medication at the time of the most recent follow-up; the median number of antihypertensive medications is 2 (range, 1–4).

There have been no deaths in this cohort. However, 2 individuals in the cohort had siblings with aHUS who died but were not included in this study because no genetic analysis was possible; pedigrees are shown in Supplementary Figure S2 and survival curves in Supplementary Figure S5.

### Transplantation

One individual, NCL29, has received a deceased donor kidney transplant. A transplanted kidney biopsy performed 2 months after transplantation because of delayed graft function showed acute tubular necrosis. At 18 months follow-up, the creatinine level is 106 μmol/l; the urine protein/creatinine ratio is 0.06 mg/mmol; and there have been no episodes of TMA.

## Discussion

In this report, we describe the phenotypic, immunologic, and genetic characteristics of a cohort of 16 individuals with DGKE nephropathy and report long-term outcomes and response to complement-inhibiting therapy and transplantation.

In England all cases of suspected aHUS are referred to a single national aHUS center (NRCTC) to undergo rigorous diagnostic evaluation and therefore this allows disease incidence to be accurately determined. Over the period from 2013 to 2019, the incidence of complement-mediated aHUS was 0.47 per million per year and 0.009 per million per year for DGKE aHUS. A national prospective study in MPGN allowed us to determine the incidence of so-called DGKE MPGN at 0.006 per million per year.

In keeping with other published cases,^1^^,^^19^ we found that the predominant phenotype was the onset of TMA in early childhood, with nephrotic range proteinuria a striking feature. The proportion with a reported infectious trigger (21%) was lower than that reported by Azukaitis *et al.* (30%)^19^ and lower than that observed with complement-mediated aHUS (≥50%).^22^ The reporting of prodromal diarrhea (29%) was similar to that reported with complement-mediated aHUS (30%).^3^ For complement-mediated aHUS, there is a plausible mechanism to explain how infections and other events such as pregnancy might trigger disease to manifest in susceptible individuals, because these so-called triggers activate complement. However, in DGKE nephropathy we do not understand why infection might precipitate disease manifestation.

We observed a high incidence (6 of 14 individuals) of developmental disorders in our cohort, including developmental delay, learning difficulties, and autistic spectrum disorder. The significance of this is not clear, though DGKE is highly expressed in the brain^8^ and studies in *DGKE* knockout mice have suggested a role in modulating neuronal signaling pathways.^23^

We identified 1 individual who presented with nephrotic syndrome but no features of aHUS, which is in keeping with the findings of Ozaltin *et al.*^2^

Although there is a range of diagnoses in the literature on what is found in the kidney in *DGKE* mutations, from published details and images of renal biopsies it is possible to see that articles are describing similar features to different extents. In 9 biopsies reported by Lemaire *et al.*,^1^ all were said to show *chronic TMA*, defined as glomerular hypercellularity and split GBMs on light microscopy and endothelial cell swelling and GBM internal lamina rara widening without electron dense deposits on EM. Ozaltin *et al.*^2^ recruited their 9 patients because they all had an original diagnosis of MPGN, and it is not surprising that their final diagnosis was an “MPGN-like glomerular microangiopathy.” Sanchez Chinchilla *et al.*^15^ reported 2 biopsies, both with glomerular TMA not defined. Westland *et al.*^16^ had 1 patient with aHUS and 1 with subacute TMA, showing patchy doubling of GBMs. Azukaitis *et al.*^19^ reported 3 patients with TMA and 2 with “TMA/MPGN” whose original pathological diagnoses were MPGN. None of these articles commented on arterioles or arteries.

Of the 5 biopsies in our series with reports of glomerular features, 2 also with images, all had doubling of GBMs to different extents, with various amounts of mesangial hypercellularity and immune deposition. Four of these had normal arterioles and arteries, while 1 had severe hyalinosis of arterioles. The other case (NCL26) had arterial intimal thickening. All these changes are described in aHUS/TMA, including the features of subendothelial MPGN.^3^ Confusion about diagnoses in the literature and in this series is probably because various pathologists reported the renal biopsy appearances. If immunohistological findings were reported, there was a range from no deposition to deposition of all immunoproteins. A reasonable interpretation is that *DGKE* mutations give an MPGN-like appearance to different extents, with or, more often, without changes in arterioles or arteries.

We report 10 novel *DGKE* mutations that are predicted to be disease causing*. DGKE* mutations throughout the entire coding region^19^ as well as an intronic cryptic splicing mutation^14^ have been reported previously, and no genotype-phenotype correlation has been determined.^19^ The results of our genetic analyses were consistent with this, with missense mutations affecting all the different functional domains. Twenty-five percent of individuals in our cohort had compound heterozygous mutations, and in 33% of the individuals with homozygous mutations, no consanguinity was reported. The individual NCL34 is interesting in that he presented comparably late (aged 8 years) and with a relatively mild phenotype (no TMA relapses and current renal function CKD stage G1 A2). In addition, this is the only pedigree in our cohort displaying incomplete penetrance; there are 3 unaffected siblings aged between 6 and 21 years (Supplementary Figure S2D) who are homozygous for the *DGKE* variant. The variant is close to the C terminus of DGKE (Figure 3b,i), and we hypothesize that it is partially functional and therefore less deleterious, resulting in a milder phenotype.

Half (7 of 14) of individuals presenting with aHUS required dialysis at the initial presentation, but this was of short duration. Forty-six percent of those with aHUS (6 of 13) received supportive management only, but in all individuals, TMA resolved and renal function recovered, regardless of management, which is in keeping with the observations of Azukaitis *et al.*^19^ The individual with a nephrotic presentation was treated with corticosteroids and angiotensin-converting enzyme inhibition with improvement in renal function and proteinuria.

Two-thirds (64%) of those presenting with TMA experienced at least 1 relapse, which is consistent with previous reports.^19^ In all but 1 individual, all relapses occurred before the age of 5 years, and in the other individual, the final relapse occurred at the age of 8 years. That no relapses occurred after this age is interesting and might suggest a key role for DGKE in kidney development and/or function in the first few years of life, which becomes progressively less important, or is compensated for by another factor.

The international consensus recommendation is that all patients with a clinical diagnosis of primary aHUS are eligible for treatment with a complement inhibitor.^4^ However, a subset of individuals with aHUS do not respond to complement-inhibiting therapy (e.g., *INF2*^24^ and *MMACHC*^25^) and are likely to have a complement-independent mechanism of TMA. The mechanism of DGKE-mediated aHUS is yet to be clearly defined. In previously published cases of DGKE aHUS, evidence of complement activation (low serum C3 or increased sC5b-9 levels) was reported in nearly one-third of patients.^19^ Two individuals with low C3 levels were said to have a good response to plasma infusions,^16^ and 1 individual with a concomitant *C3* mutation was reported to respond to plasma infusions and then eculizumab.^15^ The terminal complement inhibitor eculizumab functionally blocks C5^26^ and is recommended as the first-line treatment of aHUS that is presumed to be complement mediated.^3^ In total, the outcomes in 6 individuals who received eculizumab therapy either at the initial presentation (*n* = 3) or later in the clinical course (*n* = 3) have thus far been published, with no or transient response being reported in 4 cases and relapses while on treatment occurring in 2 individuals.^19^ In our cohort, low C3 levels were observed in 3 individuals. We report our experience with eculizumab in 6 individuals with DGKE aHUS. Two individuals from outside the United Kingdom continue on eculizumab maintenance therapy; relapse has occurred in 1 while on eculizumab, and both have CKD with proteinuria (albuminuria category A3). In the United Kingdom, we have withdrawn eculizumab in 2 individuals in whom it was commenced before the genetic diagnosis of DGKE aHUS on the assumption that they had complement-mediated aHUS. A TMA relapse occurred 6 weeks after withdrawal in 1 individual (aged 6 months), but this resolved spontaneously, and no relapses have occurred in the other individual (aged 9 years at the time of withdrawal). Both are well at 16 months after withdrawal, with CKD stage G1. In the United Arab Emirates, eculizumab was withdrawn in 1 individual 3 years ago, at the age of 8 years, with no relapse episodes. One individual (NCL29), who developed ESRD and received a kidney transplant, was treated with eculizumab for 12 months when he had progressive CKD with no appreciable clinical response, and it was withdrawn when he was found to have a *DGKE* mutation. Given that the natural history of DGKE aHUS is that relapses occur in the first 5 years of life, it is not surprising that an individual experienced a relapse after eculizumab was withdrawn at the age of 6 months.

There is insufficient evidence to define whether complement may have any role in the pathogenesis of DGKE aHUS; however, given the numerous recurrences of aHUS on patients on eculizumab, the terminal pathway is clearly not obligatory. It is unclear whether the occasional reports of low C3 or C4 levels suggesting coexistent complement activation reflects a disease-modifying role or whether it is simply a bystander effect.^26^

The predominant DGKE aHUS phenotype of TMA relapses and persistent proteinuria and progression to CKD has been described by Azukaitis *et al.*,^19^ and our data are consistent with this, with CKD and proteinuria, nonvisible hematuria, and hypertension being observed in most cases. However, we report better long-term outcomes than previously described. In our cohort, 2 individuals (siblings) (12.5%) have progressed to ESRD >20 years after the initial diagnosis as compared with 22% of individuals reaching ESRD at a median age of 12 years in the summary cohort reported by Azukaitis *et al.*,^19^ and the 2 individuals with the longest follow-up duration (31 and 45 years) in our cohort have CKD stage G2 and G3b by glomerular filtration rate category. A Kaplan-Meier curve comparing and combining our data with those published by Azukaitis *et al.*^19^ (total of 59 individuals) is shown in Supplementary Figure S5D.

The published literature reports the outcomes in 5 kidney transplant recipients, 4 with DGKE aHUS and 1 with so-called DGKE MPGN, with no recurrence of disease.^19^ We report a further individual with DGKE aHUS and successful kidney transplantation with no recurrence.

In summary, *DGKE* mutations display genetic pleiotropy, with the phenotype comprising pathological features of MPGN to different extents, and clinical features of aHUS, nephrotic syndrome, or both. The pathophysiological mechanisms of DGKE nephropathy have not yet been defined. DGKE aHUS presents in early childhood with concurrent nephrotic range proteinuria, and the natural history is one of aHUS relapses occurring only in the first few years of life. Progression to CKD with persistent proteinuria, nonvisible hematuria, and hypertension is common. Some individuals will progress to ESRD, though most do not, and predictors of progression have not yet been identified. In all individuals in our cohort, the initial TMA episode resolved regardless of management. We did not identify evidence to suggest a significant role for complement in DGKE aHUS, and we have withdrawn eculizumab in 4 individuals. Although the optimal treatment of DGKE nephropathy is not yet clear, our experience is that complement-inhibiting therapy is not beneficial and we suggest supportive management. In those individuals with DGKE aHUS in whom eculizumab has already been instituted, we recommend that it can safely be withdrawn. This ensures that these individuals are not unnecessarily exposed to the potential complications associated with complement-inhibiting therapy and has favorable economic implications.^26^ The likelihood of relapses occurring depends on the age at which eculizumab is withdrawn, with relapses only being observed in the first few years of life. DGKE is an intracellular protein, and there are no reports of DGKE nephropathy recurrence after transplantation (6 cases reported as of October 2019); therefore, individuals who progress to ESRD should undergo kidney transplantation without the need for eculizumab.

## Methods

### Patients

Patients were identified by prospective testing of incident patients with a diagnosis of aHUS or MPGN referred to the United Kingdom National Atypical Haemolytic Uremic Syndrome Service (2013–2019) or UK MPGN pediatric RaDaR (2012–2015), respectively, and also by retrospective testing of patients in the NRCTC database with a diagnosis of aHUS but no known genetic/autoimmune cause. The clinical care teams looking after patients are based in the United Kingdom (NCL25, NCL28, NCL30, NCL33, NCL34, NCL36, NCL37, and NCL39), the United Arab Emirates (NCL31, NCL32, and NCL35), the United States (NCL27 and NCL29), Brunei (NCL26), and New Zealand (NCL38). Patient identification numbers were allocated according to time of referral to our center; they begin at NCL25 to maintain consistency with our previously published study on factor H autoantibody–associated aHUS.^27^ Six patients had a renal biopsy (NCL25, NCL26, NCL27, NCL29, NCL34, and NCL37).

The pedigree of patient NCL26 was previously published before the genetic analysis.^28^ Some clinical data for patients NCL31, NCL32, and NCL35 have previously been published.^29^

The incidence rates for complement-mediated aHUS and DGKE aHUS were calculated using the estimated population of England according to the Office for National Statistics.^30^ The calculation of the incidence of complement-mediated aHUS included patients referred to the NRCTC with TMA in which the following conditions were excluded using genetic and serological analysis or on the basis of clinical context: thrombotic thrombocytopenic purpura, shiga toxin–producing *E coli* HUS, disseminated intravascular coagulation, pneumococcal HUS, HIV, drug-induced TMA, severe hypertension–associated TMA, cobalamin C deficiency–associated TMA, malignant neoplasm–associated TMA, bone marrow transplantation–associated TMA, *de novo* TMA after solid organ transplantation, glomerular disease–associated TMA, and autoimmune disease–associated TMA.^3^ The number of incident patients with complement-mediated aHUS in England in the 6 years from April 2013 to April 2019 is 157 (114 adults and 43 children).

The study was approved by the Northern and Yorkshire Multi-centre Research Ethics Committee and informed consent obtained in accordance with the Declaration of Helsinki.

### Renal function

Renal function was classified by glomerular filtration rate and albuminuria categories according to the Kidney Disease: Improving Global Outcomes recommendations.^31^

The estimated glomerular filtration rate was calculated as follows: for children (<18 years), the Schwartz formula was used: estimated glomerular filtration rate (ml/min per 1.73 m^2^) = [0.55 × height (cm) × *K* (constant)]/serum creatinine level (μmol/l) × 0.0113 (correction factor for mg/dl); in the first year of life, for preterm babies, *K* = 0.33 and for full-term infants, *K* = 0.45; for infants and children between ages of 1 and 12 years, *K* = 0.55; and for adolescent boys, *K* = 0.7. For adults, the Chronic Kidney Disease Epidemiology Collaboration equation was used: 141 × min(*S*_cr_/*κ*, 1)^*α*^ × max(*S*_cr_/*κ*, 1)^−1.209^ × 0.993^Age^ × 1.018 [if female] × 1.159 [if black], where *S*_cr_ is serum creatinine level in mg/dl, *κ* is 0.7 for females and 0.9 for males, *α* is −0.329 for females and −0.411 for males, min indicates the minimum of *S*_cr_/*κ* or 1, and max indicates the maximum of *S*_cr_/*κ* or 1.

### Complement assays

C3 and C4 levels were measured by rate nephelometry (Beckman Coulter Array 360, Beckman Coulter, High Wycombe, UK). The normal range for C3 levels was 0.68–1.38 g/l and for C4 levels 0.18–0.60 g/l. The factor H autoantibody consensus assay was performed as previously described.^32^

### Genetic analysis

Mutation screening of *CFH*,^33^ *CFI*,^34^ *CFB*,^35^ *CD46*,^36^ and *C3*^37^ was undertaken using Sanger sequencing, as previously described. Screening for chromosomal rearrangements affecting *CFH*, *CFHR1*, *CFHR2*, *CFHR3*, *CFHR4*, *CFHR5*, *CFI*, and *CD46* was undertaken using multiplex ligation–dependent probe amplification, as previously described.^38^^,^^39^ In familial cases (NCL25, NCL26, NCL27, and NCL29) whole exome sequencing was undertaken as previously described.^24^ Sanger sequencing as previously described^24^ was used to confirm DGKE mutations identified by whole exome sequencing and for routine screening.

Note: Analysis of exon 5 includes 45 bases of intron 5 to cover the region of the known cryptic splicing mutation c.888+40A>G.^14^ The nomenclature was based on accession number NM_003647.2, where the A of the ATG codon is nucleotide 1.

### Sanger sequencing analysis

Sanger sequencing analysis was performed using Sequencher v5.0 (Gene Codes Corporation, Ann Arbor, MI). The reference DGKE sequence was obtained from the Ensembl genome browser (EMBL-EBI): human: GRCh38.p10; transcript: DGKE-201 ENST00000284061.7.^40^

### RNA studies

RNA was extracted from peripheral blood lymphocytes by NewGene Limited (Newcastle upon Tyne, UK). cDNA was generated using the Invitrogen SuperScript IV First-Strand Synthesis System for reverse transcriptase polymerase chain reaction (Invitrogen, Carlsbad, CA). Primers were designed using an in-house primer design and specificity assessment tool, PrimerSNP, to amplify exons 2 to 5 (c.465-2A>G) and exons 9 to 12 (c.1524+2T>C) (see Supplementary Figure S6). Polymerase chain reaction of cDNA was performed under the following conditions: 20.5 μI lmmoRed (lmmoRed Taq mix working concentration 0.32 mM cresol red, 20% sucrose, 0.32 mM deoxynucleotides (dNTPs), 1.5× NH_4_ buffer, 2.5 mM MgCl_2_, 1 U of Immolase Taq; Bioline Reagents Ltd., London, UK), 2.5 μI forward and reverse primers (10 μM), 2 μl of cDNA; cycling conditions: 1 × 95 °C for 10 minutes, 35 × (94 °C for 1 minute, 60 °C for 1 minute, 72 °C for 1 minute), and 1 × 72 °C for 20 minutes. Products were run on a 2% ethidium bromide–stained agarose gel in tris-acetate-ethylenediaminetetraacetic acid buffer at 100 V for 3 hours.

### Amino acid conservation alignment

Amino acid conservation alignment was evaluated using the University of California Santa Cruz Genome Browser on Human Feb. 2009 (GRCh37/hg19) Assembly and BLAST-Like Alignment Tool search tool. Of the Multiz alignments of 100 vertebrates, 13 were selected: chimp, gorilla, orangutan, rhesus monkey, mouse, rat, rabbit, dolphin, dog, opossum, platypus, chicken, and zebrafish.

### *In silico* analysis

The pathogenic potential of the novel missense *DGKE* mutations was evaluated using Alamut Visual 2.10 Interactive Biosoftware (Rouen, France): build: GRCh38; transcript: NM_003647.2. Alamut automatically computes missense predictions using Align GVGD, SIFT, and MutationTaster and incorporates the Splicing Prediction Module. Minor allele frequencies are reported using NHLBI Exome Sequencing Project variants (http://evs.gs.washington.edu/EVS/).

### Statistical analyses

Patient characteristics were examined using descriptive statistics for continuous variables (mean, median) and categorical variables (number, percentage). Laboratory data are presented as mean (range). Renal survival was examined using Kaplan-Meier analysis (IBM Statistical Package for Social Sciences [SPSS], IBM Corp., Armonk, NY).

### Protein modeling

Protein modeling was undertaken as previously described.^24^

Phyre2 was used to generate an approximate protein structure by using the inputted amino acid sequence of DGKE (NP, amino acids 1–) utilizing the intensive modeling mode.^41^ Protein domain boundaries for DGKE were taken from Pfam.^42^ Three-dimensional protein structures were manipulated using PyMOL.^43^

The schematic representation of DGKE shown in Figure 3b was produced using the Simple Modular Architecture Research Tool protein domain annotation resource.^44^

## Disclosure

SJ, EKSW, NSS, and DK have received honoraria for consultancy work from Alexion Pharmaceuticals. SJ has received honoraria from Novartis and is a member of the Alexion Global aHUS Registry Scientific Advisory Board. KJM is a scientific consultant for Gemini Therapeutics. DK is a director of and scientific advisor to Gyroscope Therapeutics and has received advisory board payments from Idorsia, Novartis, ChemoCentryx, and Apellis and has a patent (patent reference: P030973GB complement factor I) pending. EKSW has received advisory board payments from Novartis. CLH has provided recent consultancy services or advice to Freeline Therapeutics, Admirx, Gyroscope Therapeutics, Roche, and GSK. CLH has received research income from Ra Pharma. DPG has received honoraria from Alexion, Novartis, and Otsuka. All the other authors declared no competing interests.
