# Supplementary material for: Long-term outcomes and response to treatment in diacylglycerol kinase epsilon nephropathy
Source: Kidney Int. 2020 Jun;97(6):1260–74. doi: 10.1016/j.kint.2020.01.045 (PMC7242908; doi:10.1016/j.kint.2020.01.045)
Supplement: Supplementary File (PDF) [file mmc1.pdf]

Supplemental Figure 1: Individual value plots for laboratory findings

A. Proteinuria at presentation

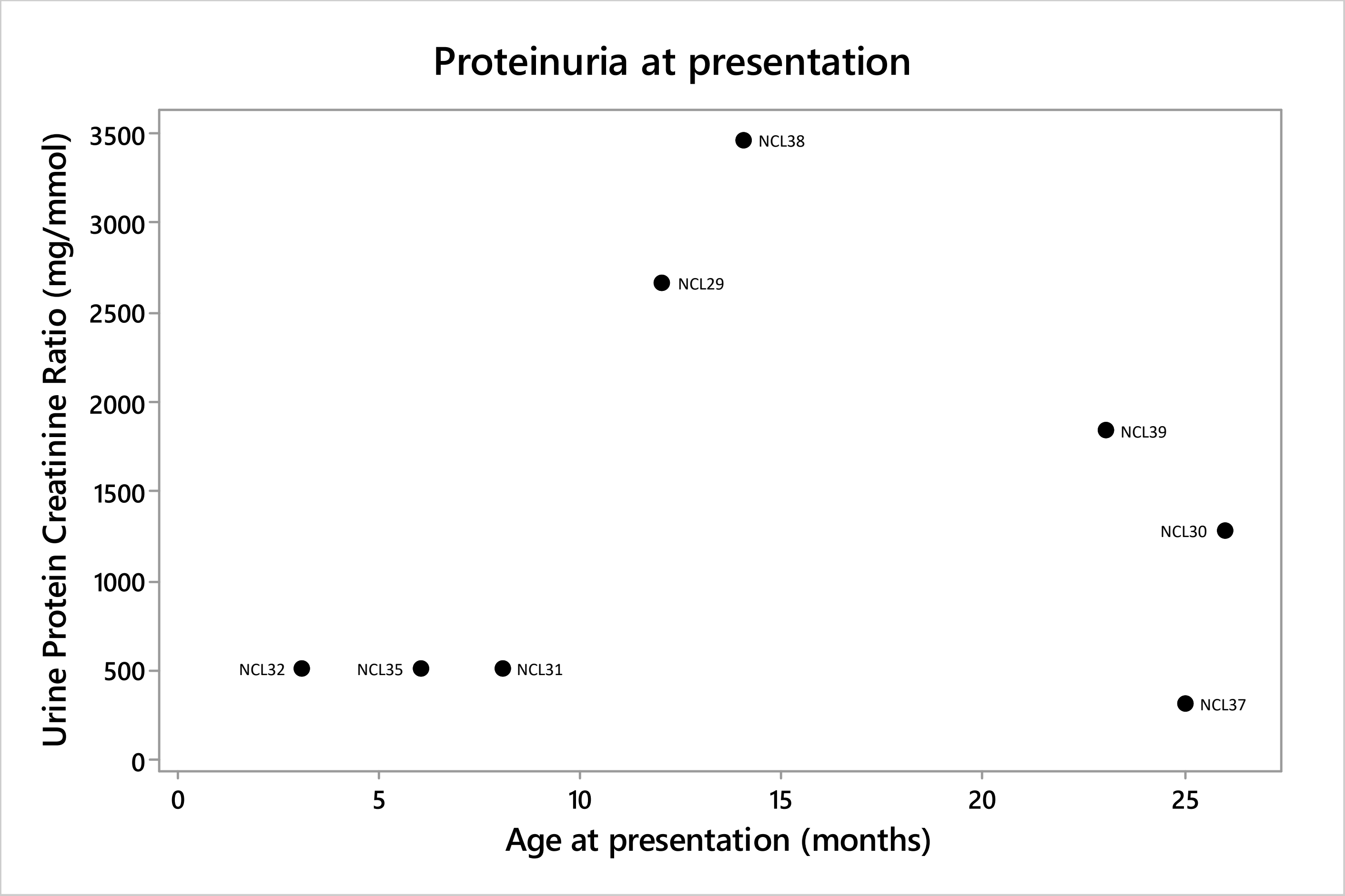

B. Creatinine at presentation

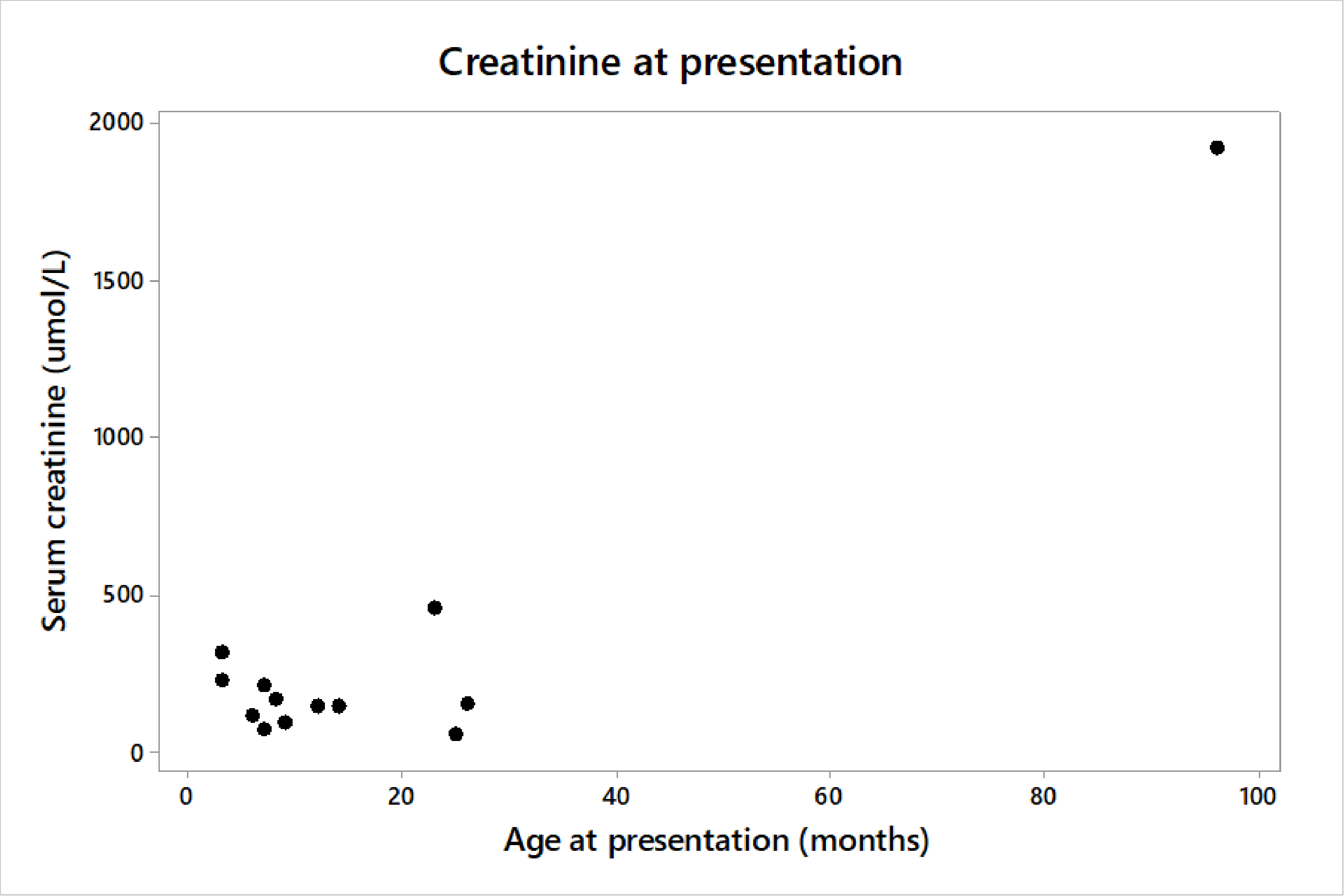

C. Complement levels at presentation

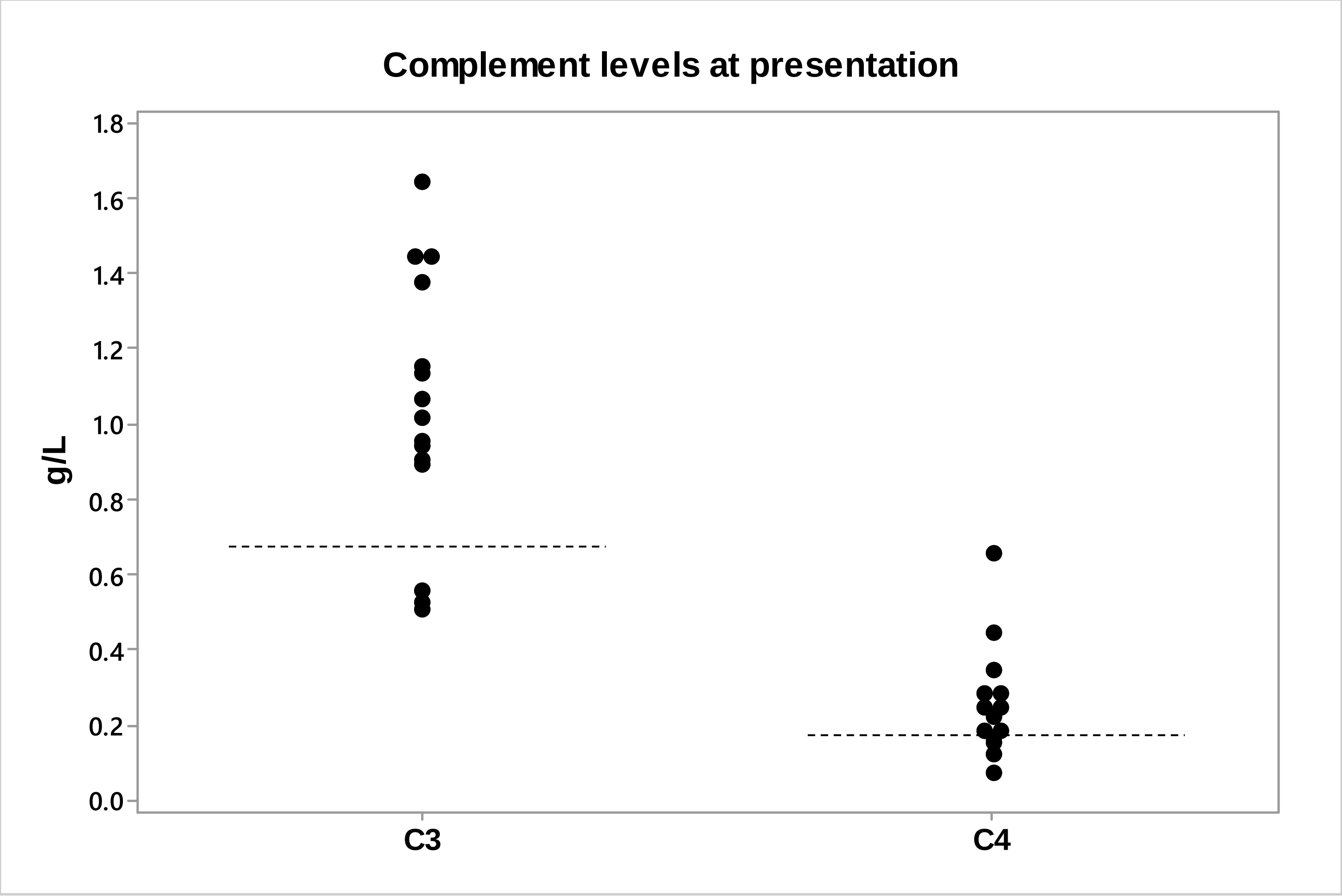

Supplemental Figure 2: Pedigrees for familial cases

A: NCL25 (II:2)

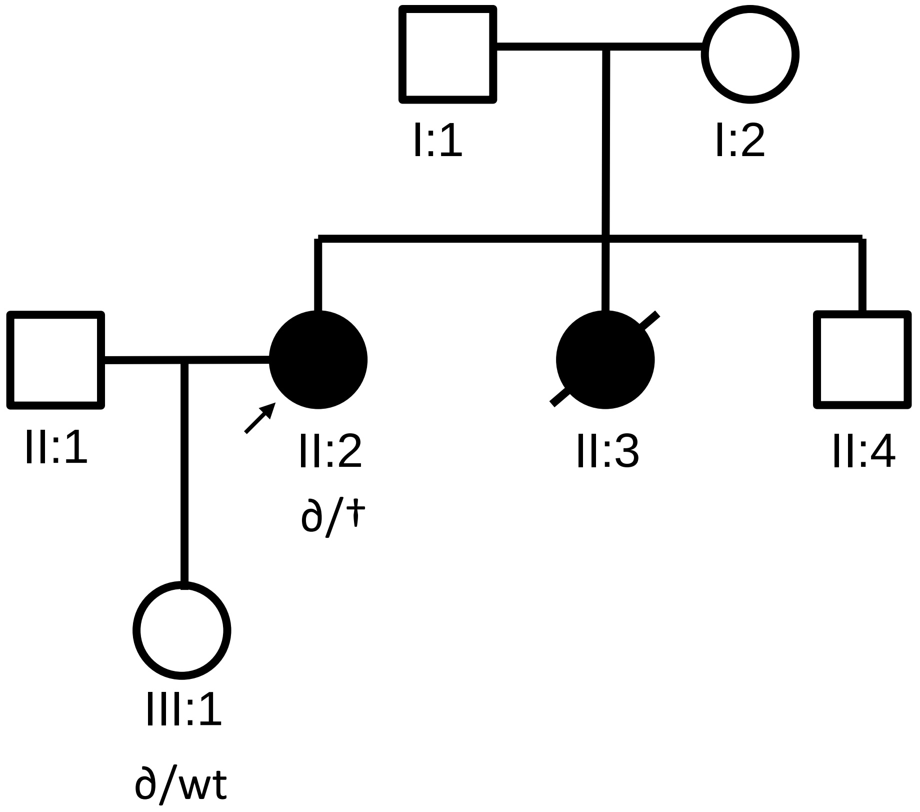

■ aHUS

∂ *DGKE* c.463A>G p.(Arg155Gly)
† *DGKE* c.1427T>C p.(Leu476Pro)
wt: wild type

B: NCL26 (II:8)

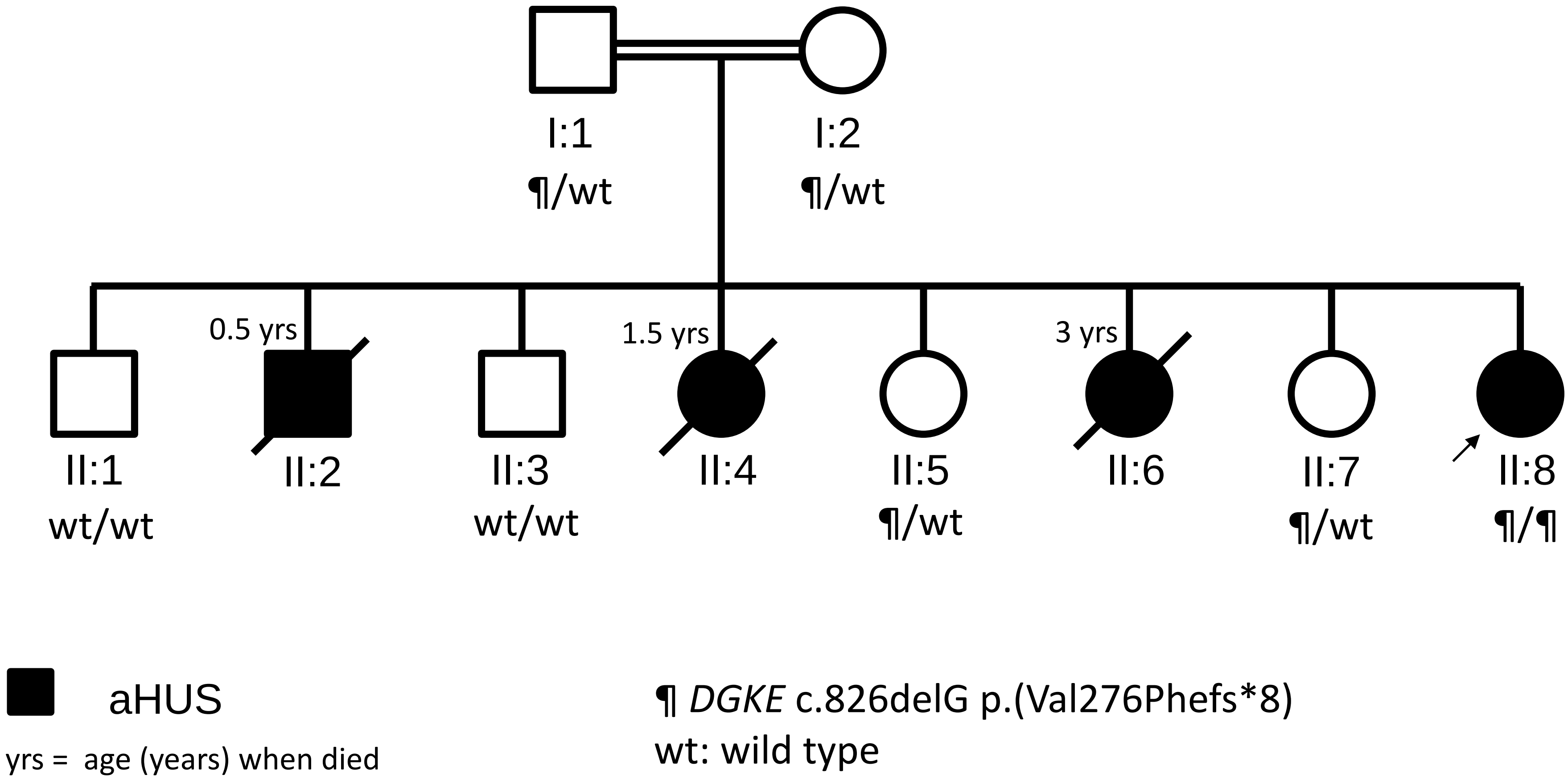

I:1
¶/wt
I:2
¶/wt
0.5 yrs
1.5 yrs
3 yrs
II:1
wt/wt
II:2
II:3
wt/wt
II:4
II:5
¶/wt
II:6
II:7
¶/wt
II:8
¶/¶
aHUS
yrs = age (years) when died
¶ *DGKE* c.826delG p.(Val276Phefs*8)
wt: wild type

NCL26 pedigree: additional clinical information

II:2

- Died aged 5 ½ months
- Death certificate recorded "Recurrent haemolytic uraemic syndrome and malignant hypertension"
- Blood tests before his death showed anaemia, kidney failure with a few fragments and burr cells, and thrombocytopaenia
- Kidney biopsy showed very large quantities of fibrillary material between the basement membranes and endothelial cells, extending into mesangial areas and also some 'formed, spherical, electron dense particles' in places; appearances were felt to be consistent with HUS

II:4

- Presented aged 3 ½ months with anaemia
- Presented aged 10 months with oedema and proteinuria; diagnosed with 'mild HUS'
- Episode of oedema after a measles vaccination
- Presented aged 18 months with febrile episode, oedema and vomiting
  - Haemolysis and AKI
  - Consent was not given for dialysis
  - Deteriorated with convulsions, anuria, and AKI
  - Then had peritoneal dialysis but remained ill with poorly controlled hypertension and heart failure
  - Subsequently had a cardiac arrest and died

II:6

- Presented aged 7 months with proteinuria and haemolysis
- HUS 'gradually came under control'
- Relapse aged 3 years: presented with vomiting and diarrhoea
  - HUS was diagnosed 10 days later, complicated by pulmonary oedema, heart failure and *E. coli* urinary tract infection
- Relapse aged 3 years 6 months
  - Consent was not given for dialysis
  - Died 1 month later

C: NCL27 (III:1) and NCL29 (III:2)

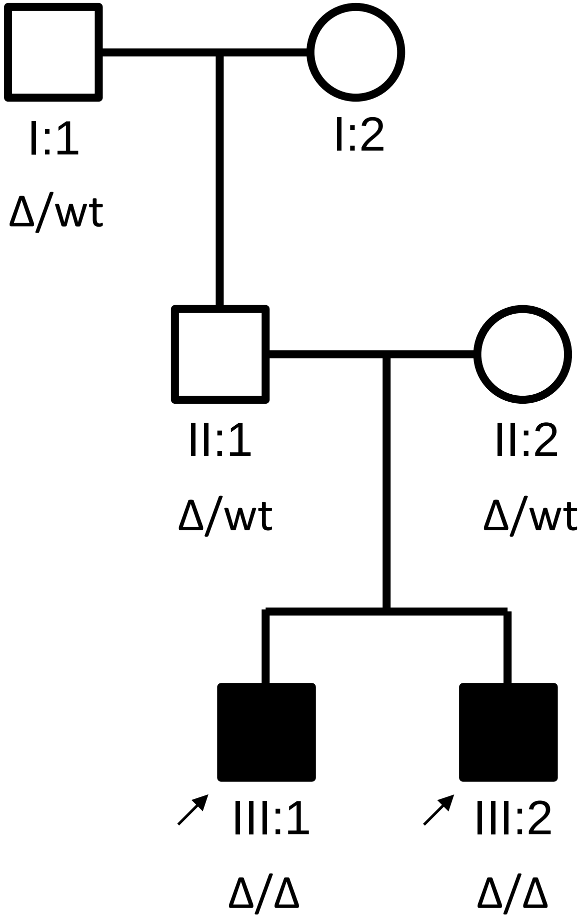

aHUS/MPGN

Δ *DGKE* c.1597A>C p.(Thr533Pro)
wt: wild type

D: NCL34 (II:4)

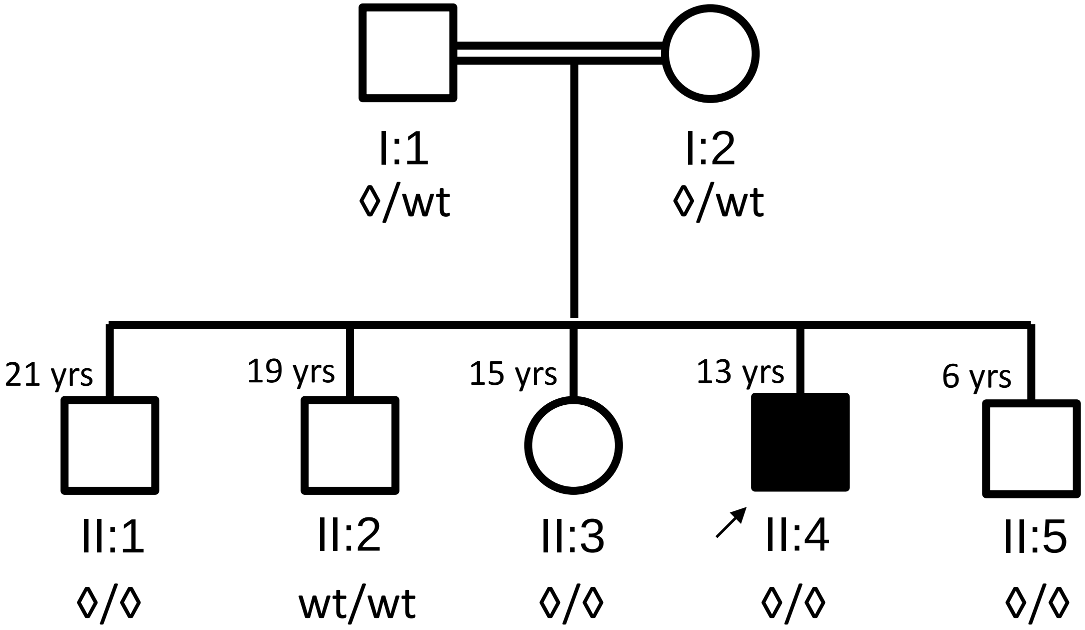

aHUS

yrs = current age (2019)

◊ *DGKE* c.1647_1650delAACA p.(T550Mfs*13)

Supplemental Figure 3: Sanger sequencing traces

A. NCL25. a. *DGKE* exon 2. b. *DGKE* exon 11.

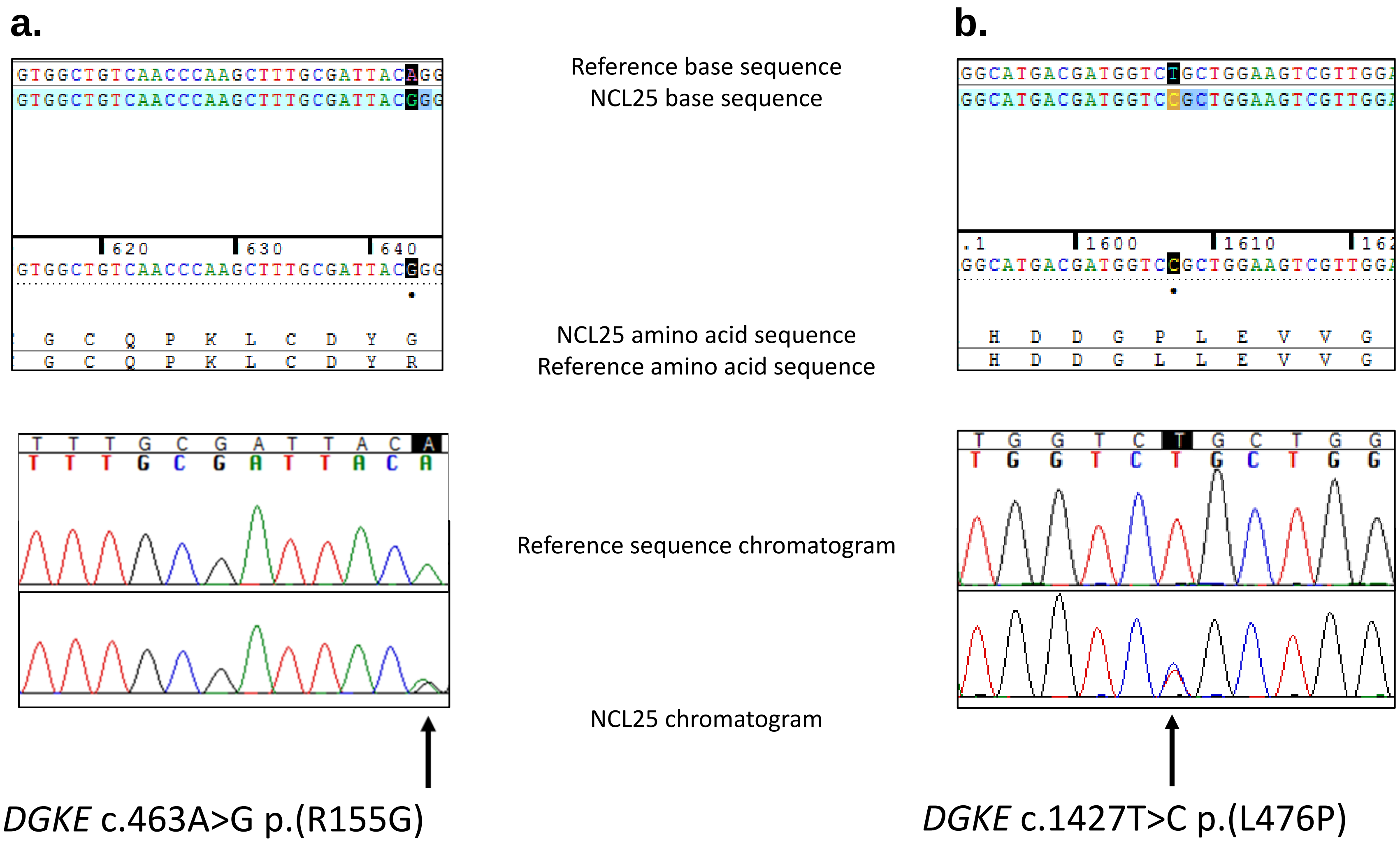

B. NCL27. *DGKE* exon 12

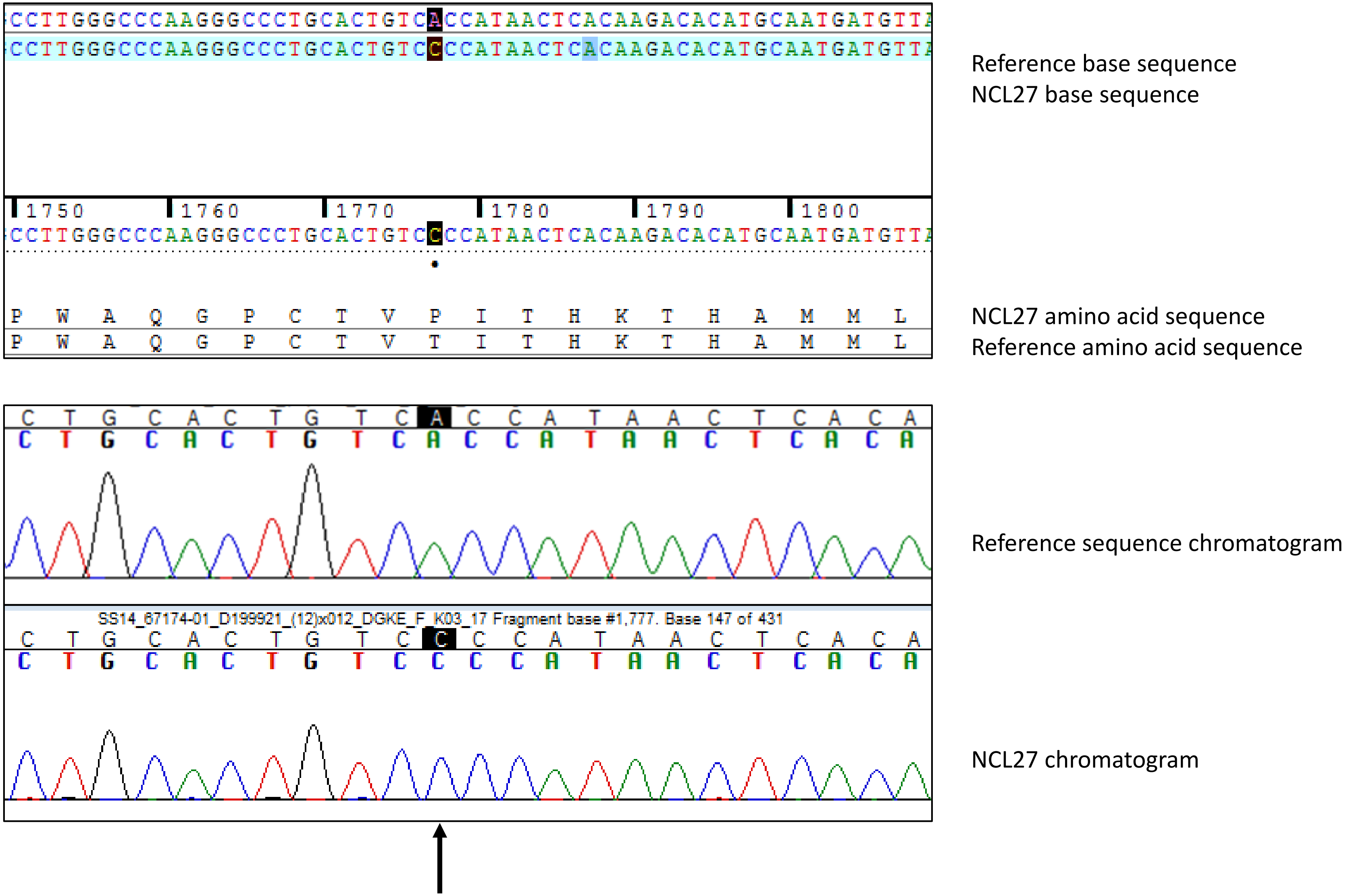

DGKE c.1597A>C p.(T533P)

C. NCL29. *DGKE* exon 12

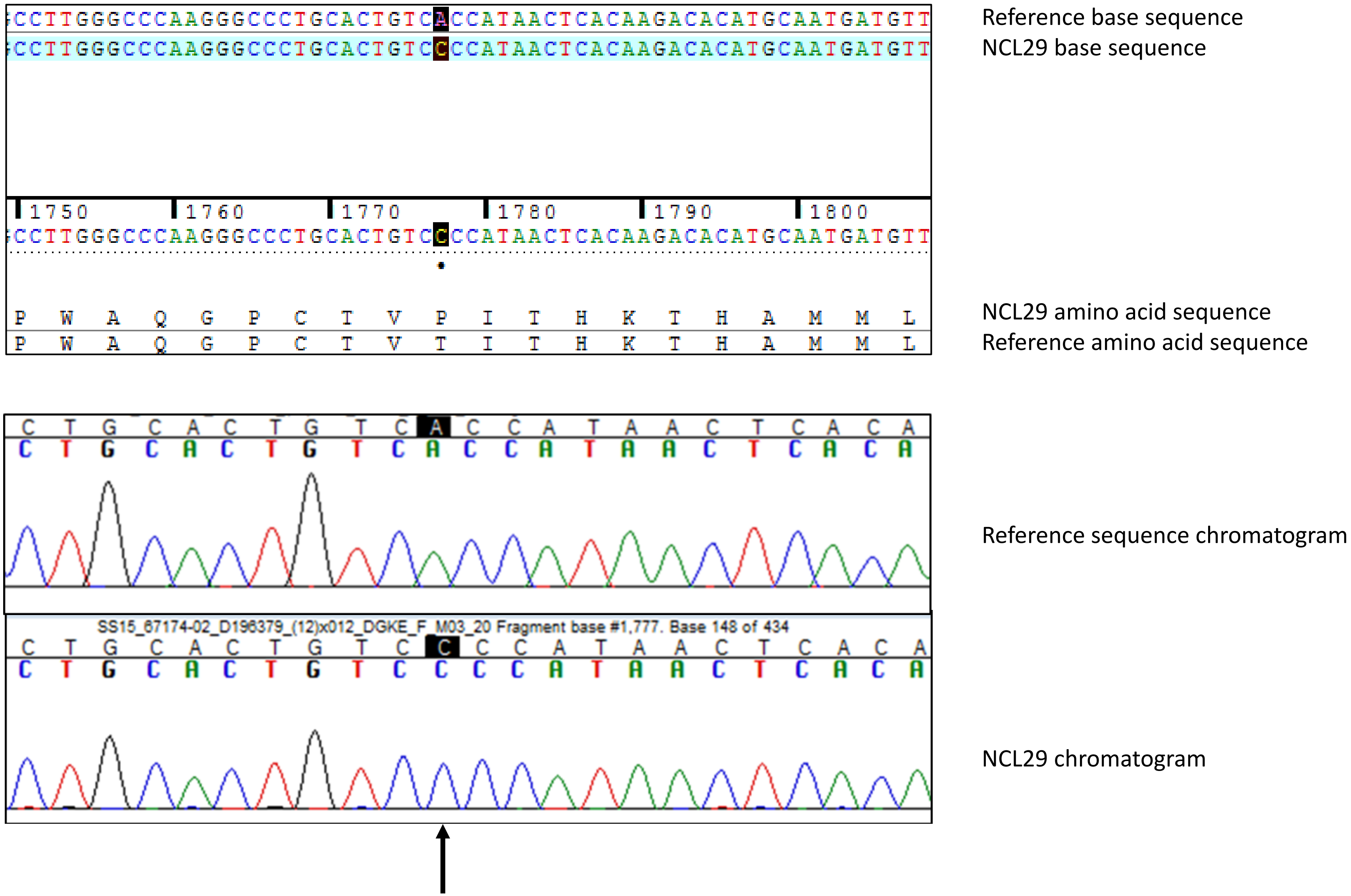

DGKE c.1597A>C p.(T533P)

D. NCL30. a. ***DGKE*** **exon 2. b.** ***DGKE*** **exon 3.**

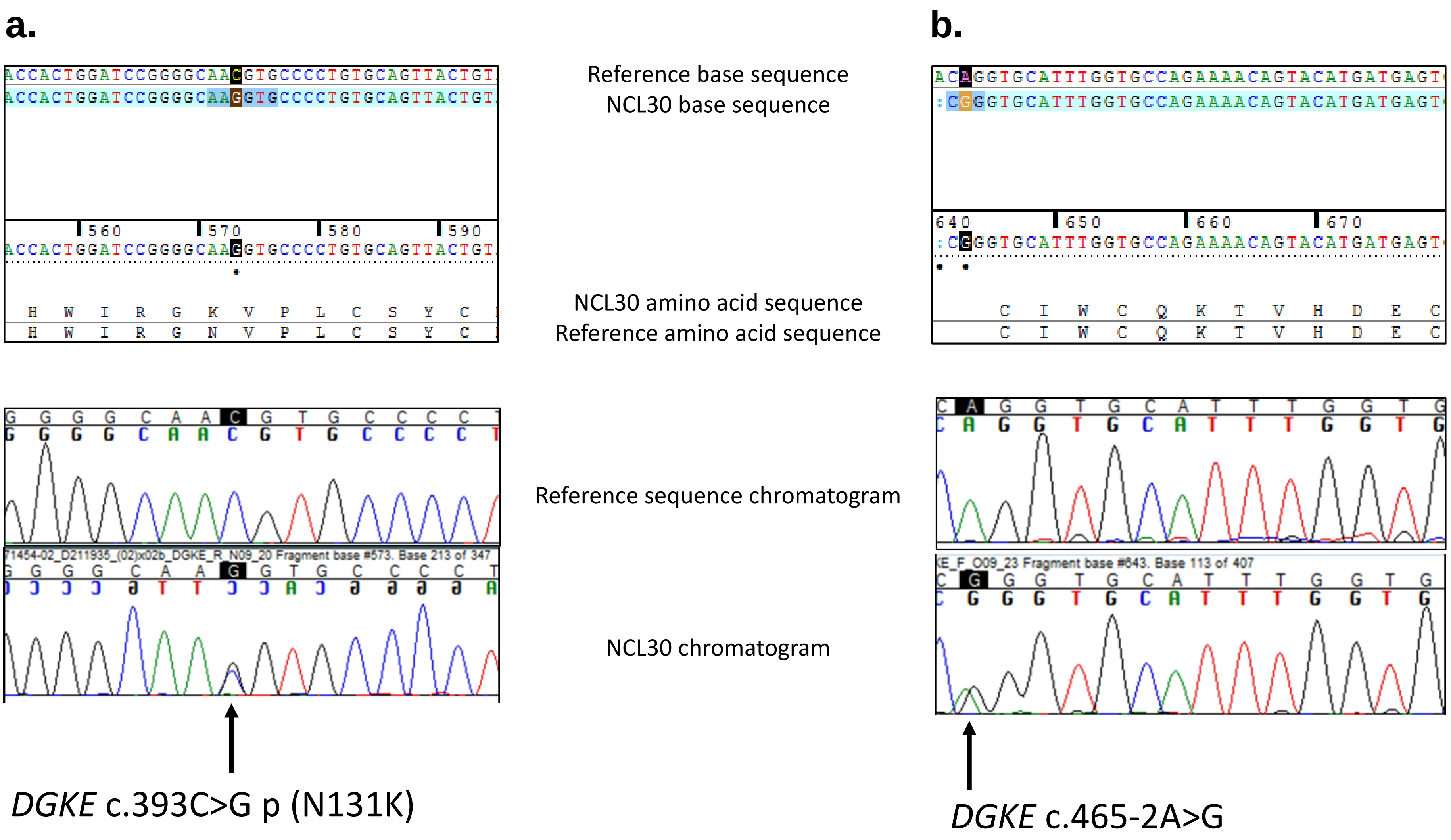

E. NCL31. *DGKE* exon 2

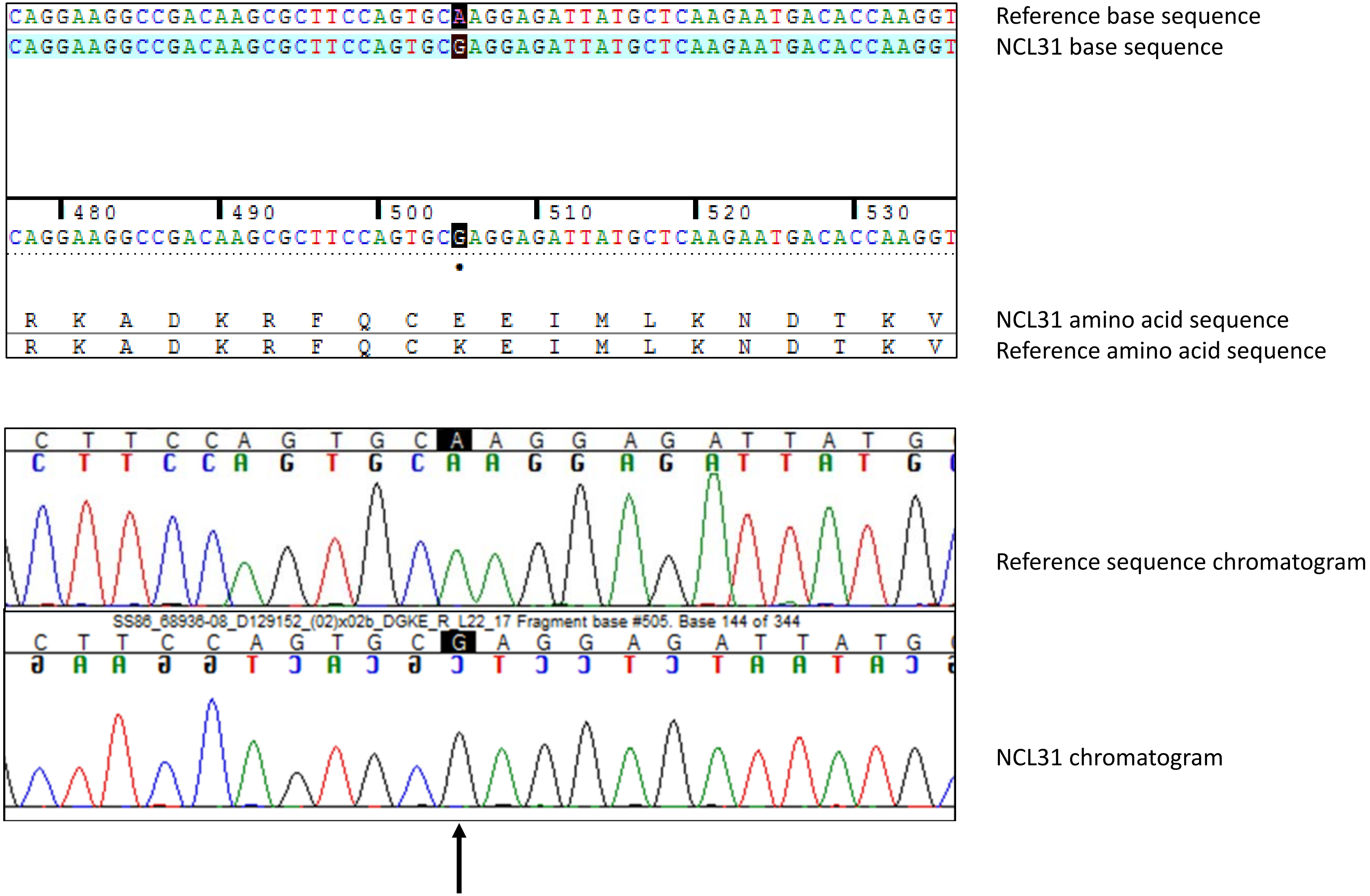

DGKE c.325A>G p.(K109E)

F. NCL33. *DGKE* exon 6

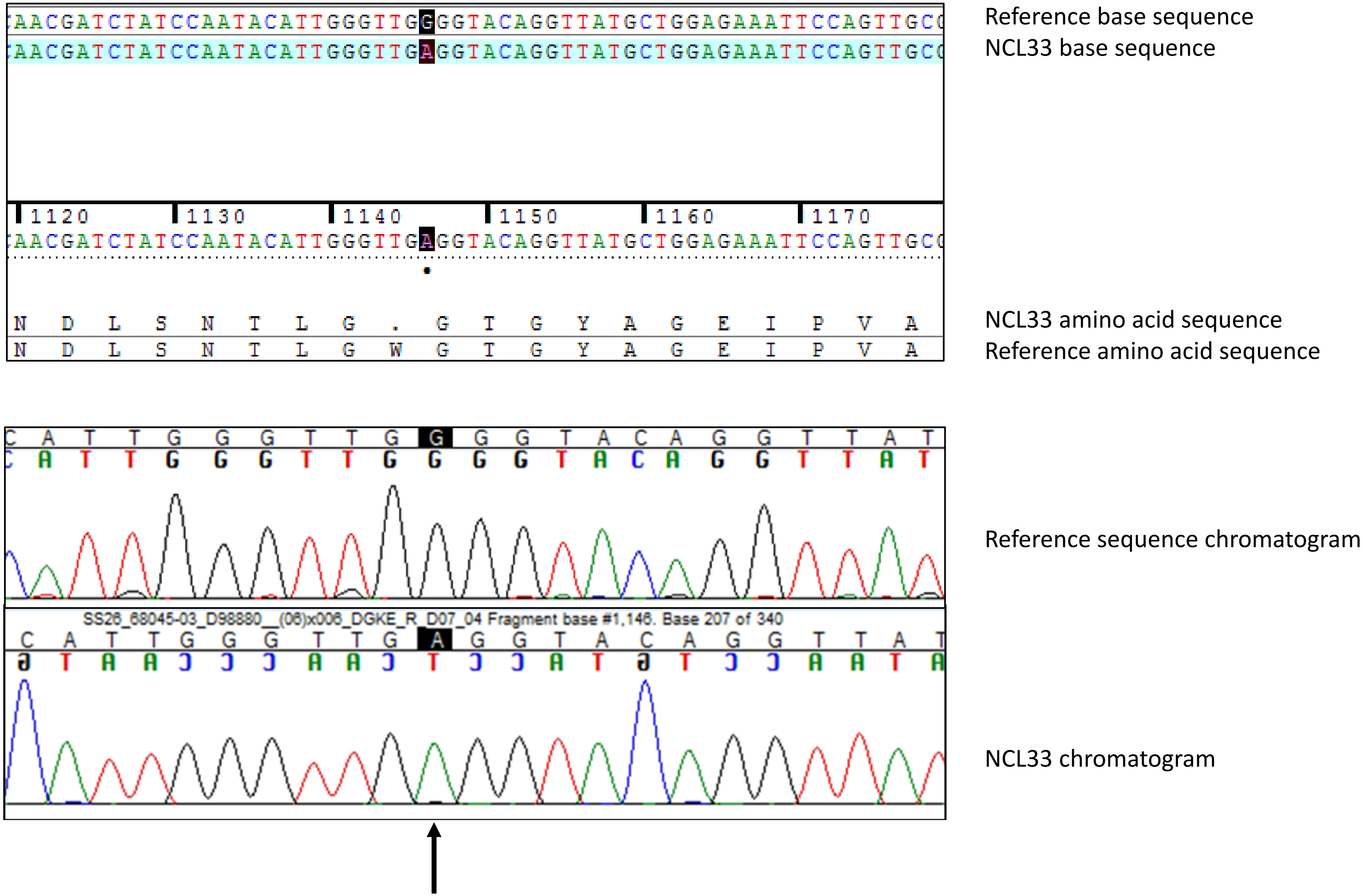

DGKE c.966G>A p.(Trp322*)

G. NCL34. *DGKE* exon 12

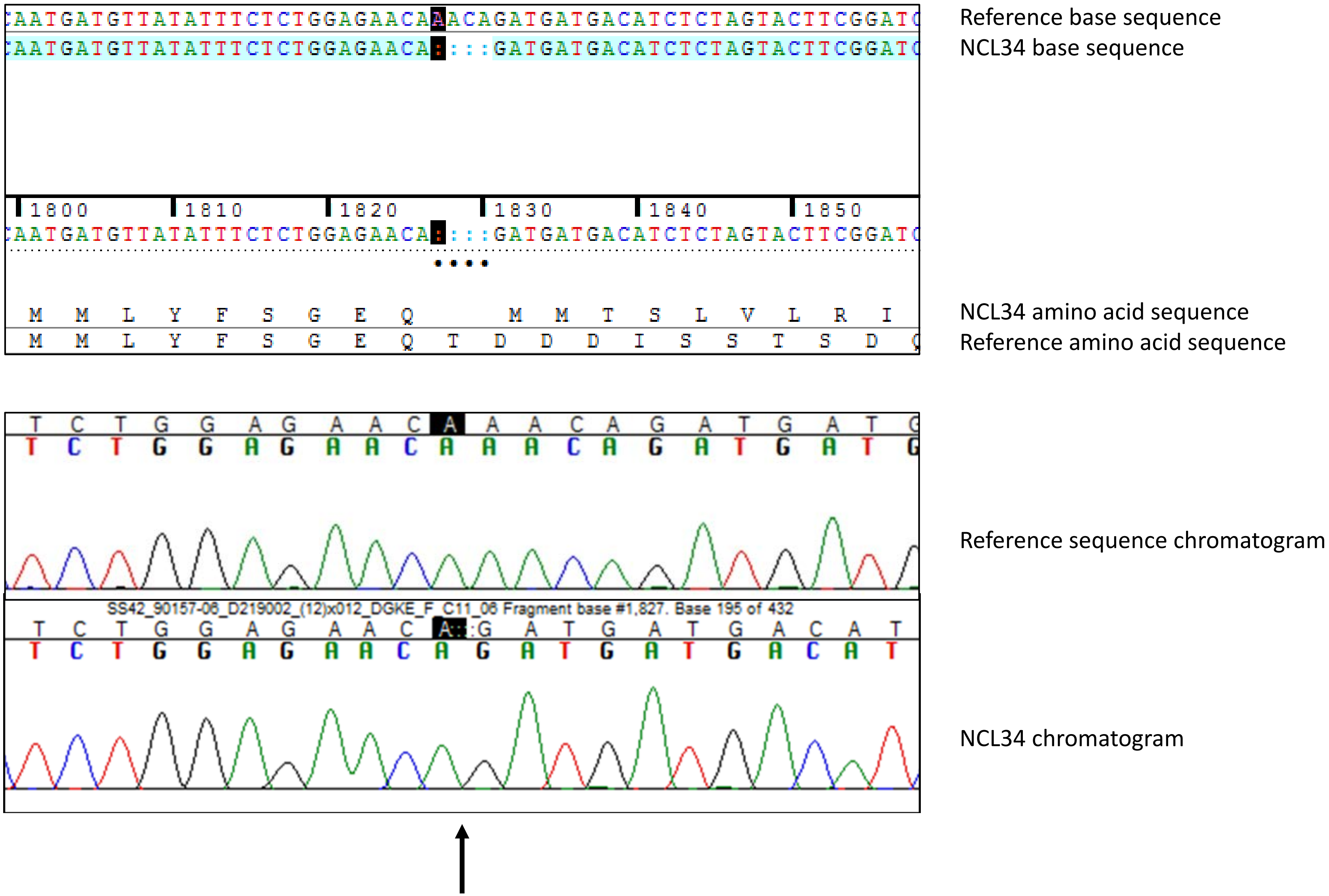

DGKE c.1647_1650delAACA p.(T550Mfs*13)

H. NCL36. a. *DGKE* exon 6. b. *DGKE* exon 11.

a.

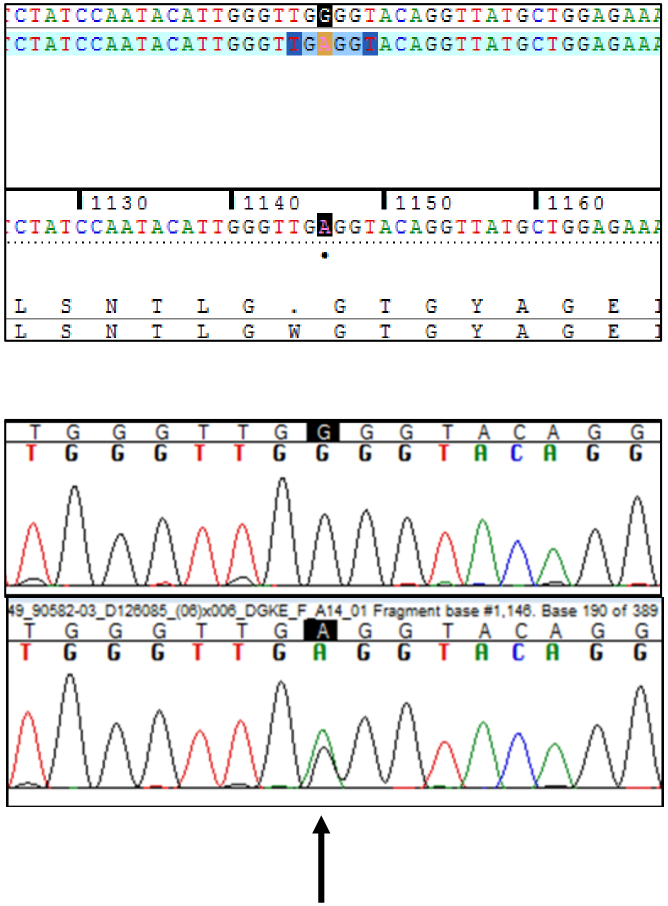

DGKE c.966G>A p.(Trp322*)

Reference base sequence

NCL36 base sequence

NCL36 amino acid sequence

Reference amino acid sequence

Reference sequence chromatogram

NCL36 chromatogram

b.

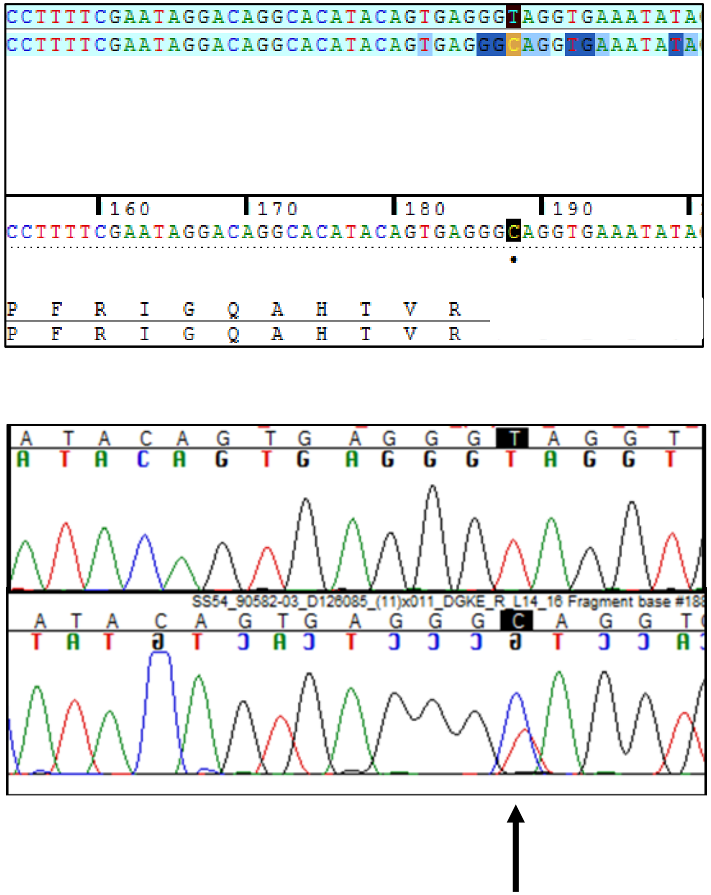

DGKE c.1524+2T>C

I. NCL37. *DGKE* exon

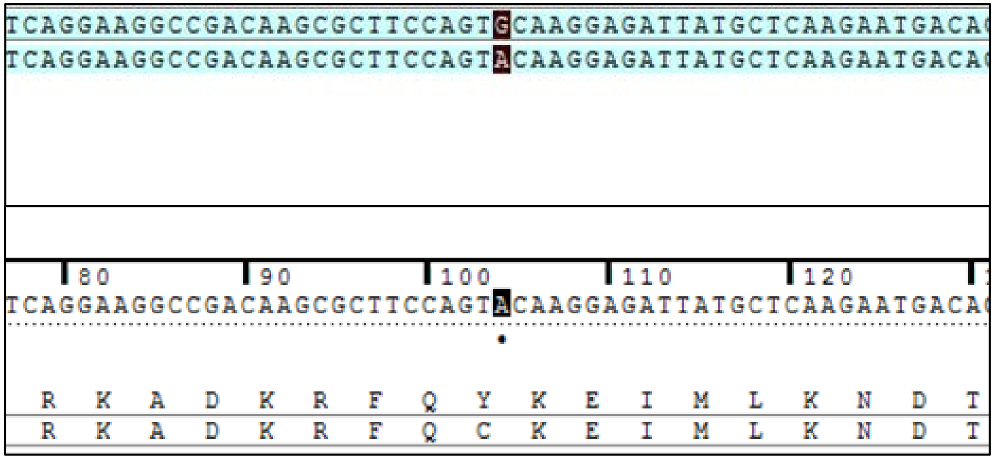

Reference base sequence
NCL37 base sequence

NCL37 amino acid sequence
Reference amino acid sequence

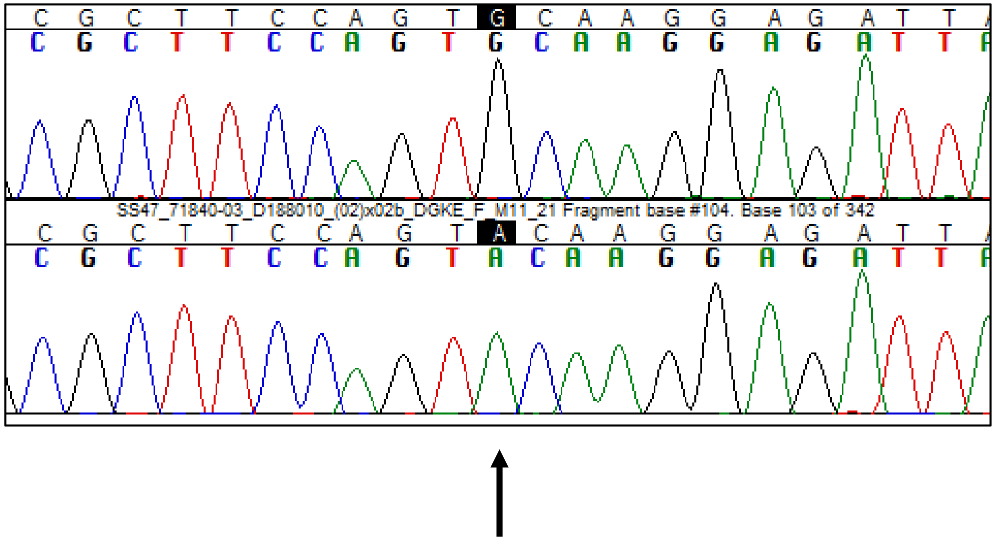

Reference sequence chromatogram

NCL37 chromatogram

DGKE c.323G>A p.(C108Y)

J. NCL38. *DGKE* exon 6

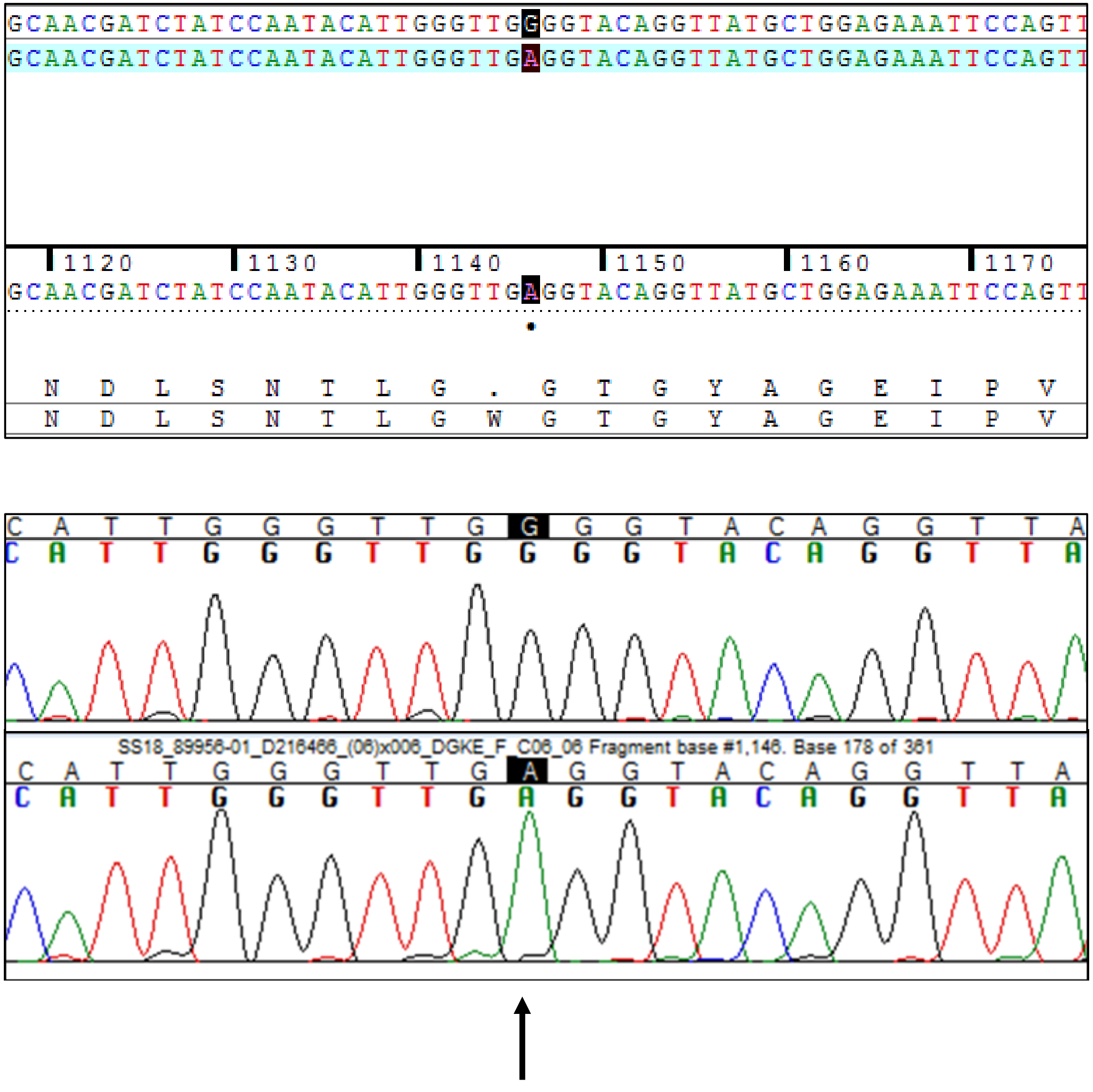

DGKE c.966G>A p.(Trp322*)

K. NCL39. a. *DGKE* exon 3. b. *DGKE* exon 6.

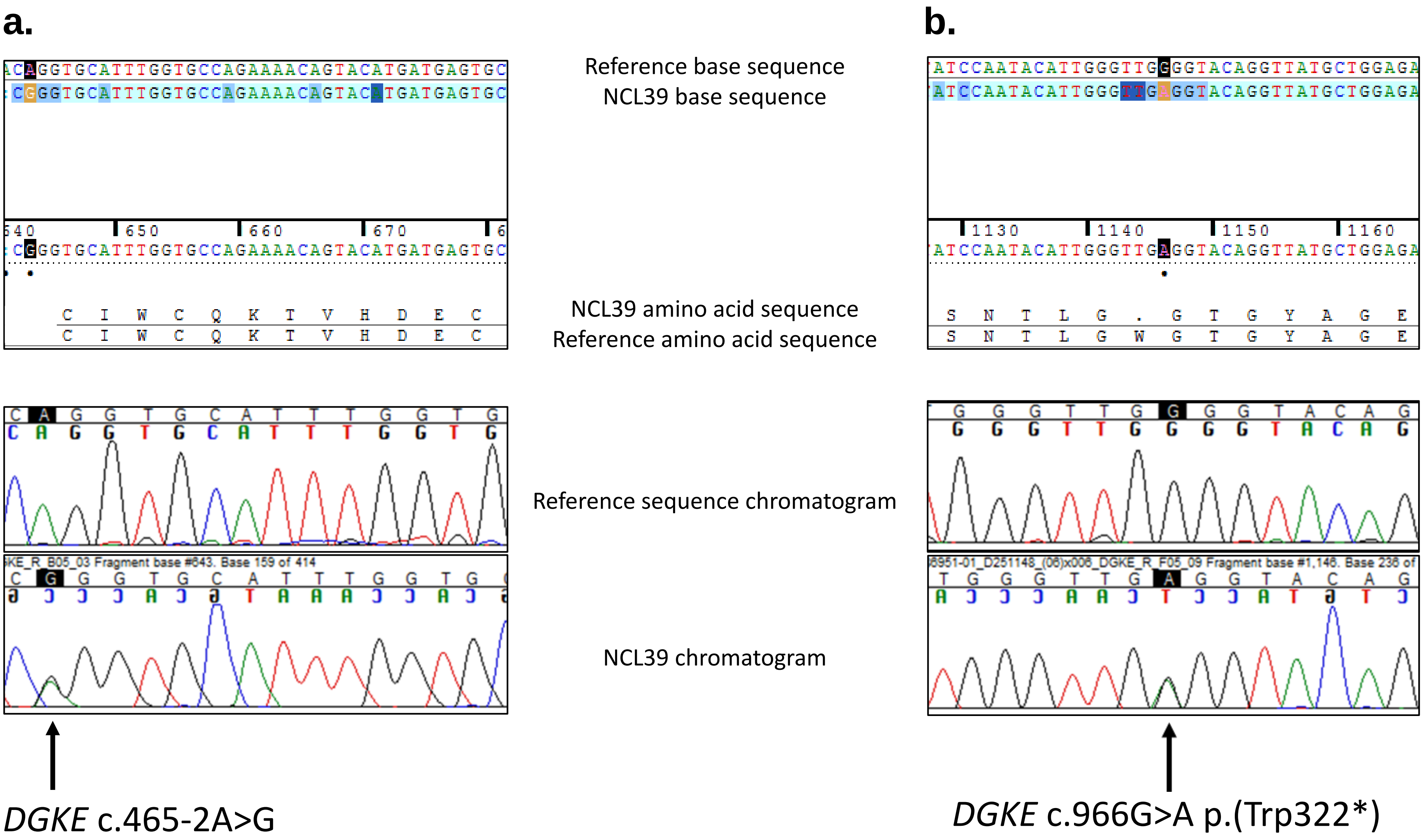

L. NCL40. *DGKE* exon 6

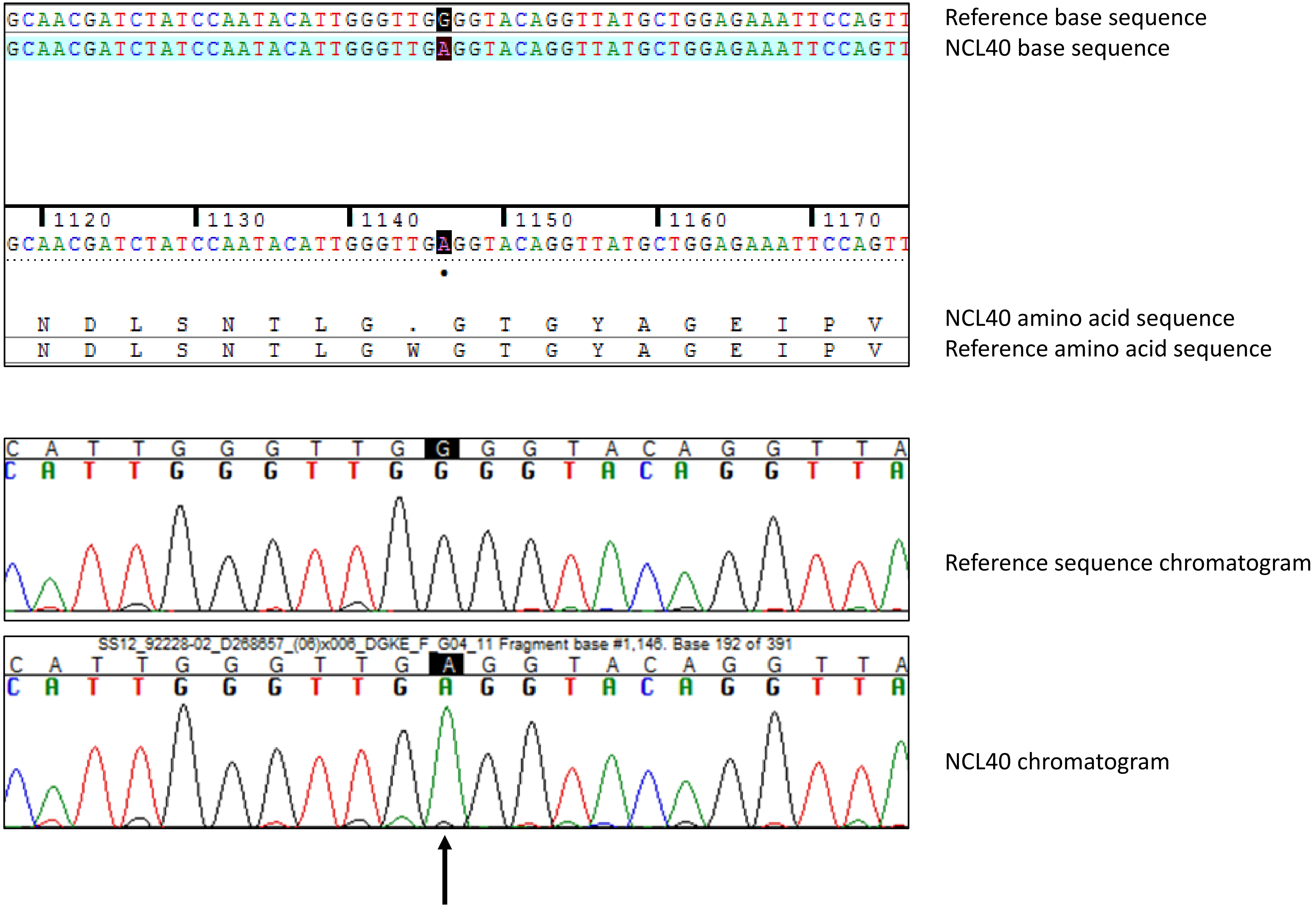

DGKE c.966G>A p.(Trp322*)

Supplemental Figure 4: DGKE amino acid conservation alignments for newly described missense mutations

A. NCL25. i. R155G. ii. L476P

i.

**Species**	**Amino acid sequence**										
**NCL25**	K<br>150	L<br>151	C<br>152	D<br>153	Y<br>154	**G**<br>**155**	C<br>156	I<br>157	W<br>158	C<br>159	Q<br>160
Human	K	L	C	D	Y	R	C	I	W	C	Q
Chimp	K	L	C	D	Y	R	C	I	W	C	Q
Gorilla	K	L	C	D	Y	-	-	-	-	-	-
Orangutan	K	L	C	D	Y	R	C	I	W	C	Q
Rhesus Monkey	K	L	C	D	Y	R	C	I	W	C	Q
Mouse	K	L	C	D	Y	R	C	I	W	C	Q
Rat	K	L	C	D	Y	R	C	V	W	C	Q
Rabbit	K	L	C	D	Y	R	C	I	W	C	Q
Dolphin	K	L	C	D	Y	R	C	I	W	C	Q
Dog	K	L	C	D	Y	R	C	I	W	C	Q
Opossum	K	L	C	D	Y	R	C	I	W	C	Q
Platypus	K	L	C	D	Y	R	C	I	W	C	Q
Chicken	K	L	C	D	Y	R	C	V	W	C	Q
Zebrafish	K	L	C	D	Y	R	C	V	W	C	Q

ii.

**Species**	**Amino acid sequence**										
**NCL25**	R<br>471	H<br>472	D<br>473	D<br>474	G<br>475	**P**<br>**476**	L<br>477	E<br>478	V<br>479	V<br>480	G<br>481
Human	R	H	D	D	G	L	L	E	V	V	G
Chimp	R	H	D	D	G	L	L	E	V	V	G
Gorilla	R	H	D	D	G	L	L	E	V	V	G
Orangutan	R	H	D	D	G	L	L	E	V	V	G
Rhesus Monkey	R	H	D	D	G	L	L	E	V	V	G
Mouse	R	H	D	D	G	L	L	E	**I**	V	G
Rat	R	H	D	D	G	L	L	E	V	V	G
Rabbit	R	H	D	D	G	L	L	E	V	V	G
Dolphin	R	H	D	D	G	L	L	E	V	V	G
Dog	R	H	D	D	G	L	L	E	V	V	G
Opossum	S	H	D	D	G	L	L	E	V	V	G
Platypus	R	H	D	D	G	L	L	E	V	V	G
Chicken	R	H	D	D	G	L	L	E	V	V	G
Zebrafish	**R**	**V**	D	D	G	L	L	E	V	V	G

B. NCL27 and NCL29. T533P

Species	Amino acid sequence										
NCL27 and NCL29	G 528	P 529	C 530	T 531	V 532	**P 533**	I 534	T 535	H 536	K 537	T 538
Human	G	P	C	T	V	T	I	T	H	K	T
Chimp	G	P	C	T	V	T	I	T	H	K	T
Gorilla	G	P	C	T	V	T	I	T	H	K	T
Orangutan	G	P	C	T	V	T	I	T	H	K	T
Rhesus Monkey	G	P	C	T	V	T	I	T	H	K	T
Mouse	G	P	C	T	V	T	I	T	H	K	T
Rat	G	P	C	T	V	T	I	T	H	K	T
Rabbit	G	P	C	T	V	T	I	T	H	K	T
Dolphin	G	P	C	T	V	T	I	T	H	K	T
Dog	G	P	C	T	V	T	I	T	H	K	T
Opossum	G	P	C	I	V	T	I	T	H	K	T
Platypus	G	P	C	T	V	T	I	T	H	K	T
Chicken	G	P	C	T	V	T	I	T	H	K	T
Zebrafish	G	P	C	T	I	T	I	T	H	K	T

C. NCL28. M1L

Species	Amino acid sequence					
**NCL36**	**L 1**	E 2	A 3	E 4	R 5	R 6
Human	M	E	A	E	R	R
Chimp	M	E	A	E	R	R
Gorilla	M	E	A	E	R	R
Orangutan	M	E	A	E	R	R
Rhesus Monkey	M	E	A	E	R	R
Mouse	M	E	G	D	Q	R
Rat	M	E	G	D	Q	R
Rabbit	M	E	G	E	R	R
Dolphin	M	E	G	E	K	R
Dog	M	E	A	E	R	R
Opossum	M	E	G	R	K	Q
Platypus	M	E	R	G	E	P
Chicken	M	L	P	E	A	A
Zebrafish	M	E	E	N	N	E

D. NCL30. N131K

Species	Amino acid sequence										
NCL30	H 126	W 127	I 128	R 129	G 130	**K 131**	V 132	P 133	L 134	C 135	S 136
Human	H	W	I	R	G	N	V	P	L	C	S
Chimp	H	W	I	R	G	N	V	P	L	C	S
Gorilla	H	W	I	R	G	N	V	P	L	C	S
Orangutan	H	W	I	R	G	N	V	P	L	C	S
Rhesus Monkey	H	W	I	R	G	N	V	P	L	C	S
Mouse	H	W	I	R	G	N	V	P	L	C	S
Rat	H	W	I	R	G	N	V	P	L	C	T
Rabbit	H	W	I	R	G	N	V	P	L	C	S
Dolphin	H	W	I	R	G	N	V	P	L	C	S
Dog	H	W	I	R	G	N	V	P	L	C	S
Opossum	H	W	I	R	G	N	V	P	L	C	S
Platypus	R	W	I	R	G	N	V	P	L	C	S
Chicken	H	W	I	R	G	N	V	P	L	C	S
Zebrafish	Q	W	V	K	G	N	V	P	L	A	S

E. NCL32. Q79P

Species	Amino acid sequence										
**NCL32**	C 74	C 75	V 76	C 77	A 78	**P 79**	H 80	I 81	L 82	Q 83	G 84
Human	C	C	V	C	A	Q	H	I	L	Q	G
Chimp	C	C	V	C	A	Q	H	I	L	Q	G
Gorilla	C	C	V	C	A	Q	H	I	L	Q	G
Orangutan	C	C	V	C	A	Q	H	I	L	Q	G
Rhesus Monkey	C	C	V	C	A	Q	H	I	L	Q	G
Mouse	C	C	V	C	A	Q	H	I	L	Q	G
Rat	C	C	V	C	A	Q	H	I	L	Q	G
Rabbit	C	C	V	C	A	Q	H	I	L	R	G
Dolphin	C	C	V	C	A	Q	H	I	L	Q	G
Dog	C	C	V	C	A	Q	H	I	L	L	G
Opossum	C	C	V	C	T	Q	H	I	L	Q	G
Platypus	C	C	L	C	A	Q	H	I	L	R	G
Chicken	C	C	V	C	A	Q	H	I	L	R	G
Zebrafish	C	C	V	C	C	Q	P	I	L	Q	G

F. NCL37. C108Y

Species	Amino acid sequence										
**NCL37**	D 103	K 104	R 105	F 106	Q 107	**Y 108**	K 109	E 110	I 111	M 112	L 113
Human	D	K	R	F	Q	C	K	E	I	M	L
Chimp	D	K	R	F	Q	C	K	E	I	M	L
Gorilla	D	K	R	F	Q	C	K	E	I	M	L
Orangutan	D	K	R	F	Q	C	K	E	I	M	L
Rhesus Monkey	D	R	R	F	Q	C	K	E	I	M	L
Mouse	D	K	R	F	P	C	K	E	I	M	L
Rat	D	K	R	F	P	C	K	E	I	M	L
Rabbit	D	R	R	F	A	C	K	E	I	V	L
Dolphin	D	K	R	F	H	C	K	E	I	M	L
Dog	D	K	R	F	P	C	K	E	I	M	L
Opossum	D	R	Q	F	Q	C	K	E	I	M	L
Platypus	D	R	R	F	P	C	K	E	I	M	L
Chicken	D	Q	H	F	L	C	K	E	I	V	M
Zebrafish	D	R	I	L	S	C	K	E	I	M	T

Supplemental Table 1: *In silico* analysis of newly described missense *DGKE* mutations

Patient	Inheritance	DGKE variant	ESP frequencies	Align GVGD	SIFT	MutationTaster
NCL25	Compound heterozygous	c.463A>G p.(Arg155Gly)¶	N/A	Class C65 (GV: 0.00 - GD: 125.13)	Deleterious (score: 0)	Disease causing (p-value: 1)
		c.1427T>C p.(Leu476Pro)¶	N/A	Class C45 (GV: 14.30 - GD: 86.59)	Deleterious (score: 0.01)	Disease causing (p-value: 1)
NCL26	Homozygous	c.826delG p.(Val276Phefs*8)¶	N/A	NA: frameshift		
NCL27	Homozygous	c.1597A>C p.(Thr533Pro)¶	N/A	Class C0 (GV: 121.10 - GD: 28.88)	Tolerated (score: 0.23)	Disease causing (p-value: 1)
NCL28	Homozygous	c.1A>T p.(Met1Leu)¶	N/A	NA: start loss		
NCL29	Homozygous	c.1597A>C p.(Thr533Pro) ¶	N/A	Class C0 (GV: 121.10 - GD: 28.88)	Tolerated (score: 0.23)	Disease causing (p-value: 1)
NCL30	Compound heterozygous	c.393C>G p.(Asn131Lys)¶	N/A	Class C65 (GV: 0.00 - GD: 93.88)	Deleterious (score: 0)	Disease causing (p-value: 1)
		c.465-2A>G ¶	N/A	NA: splice site		
NCL32	Homozygous	c.236A>C p.(Gln79Pro)¶	N/A	Class C0 (GV: 261.33 - GD: 0.00)	Tolerated (score: 0.14)	Disease causing (p-value: 1)
NCL34	Homozygous	c.1647_1650delAACA p.(Thr550Metfs*13)¶	N/A	NA: frameshift		
NCL37	Homozygous	c.323G>A p.(Cys108Tyr)¶	N/A	Class C65 (GV: 0.00 - GD: 193.72)	Deleterious (score: 0)	Disease causing (p-value: 1)

Abbreviations: DGKE, diacyl glycerol kinase Ɛ; ESP, Exome Sequencing Project; GVGD, Grantham Variation Grantham Difference; NA, not applicable; N/A, not available; SIFT, Sorting Tolerant From Intolerant.

Supplemental Figure 5: Kaplan Meier Survival Curves

A. Renal survival comparing individuals who received eculizumab with those who did not

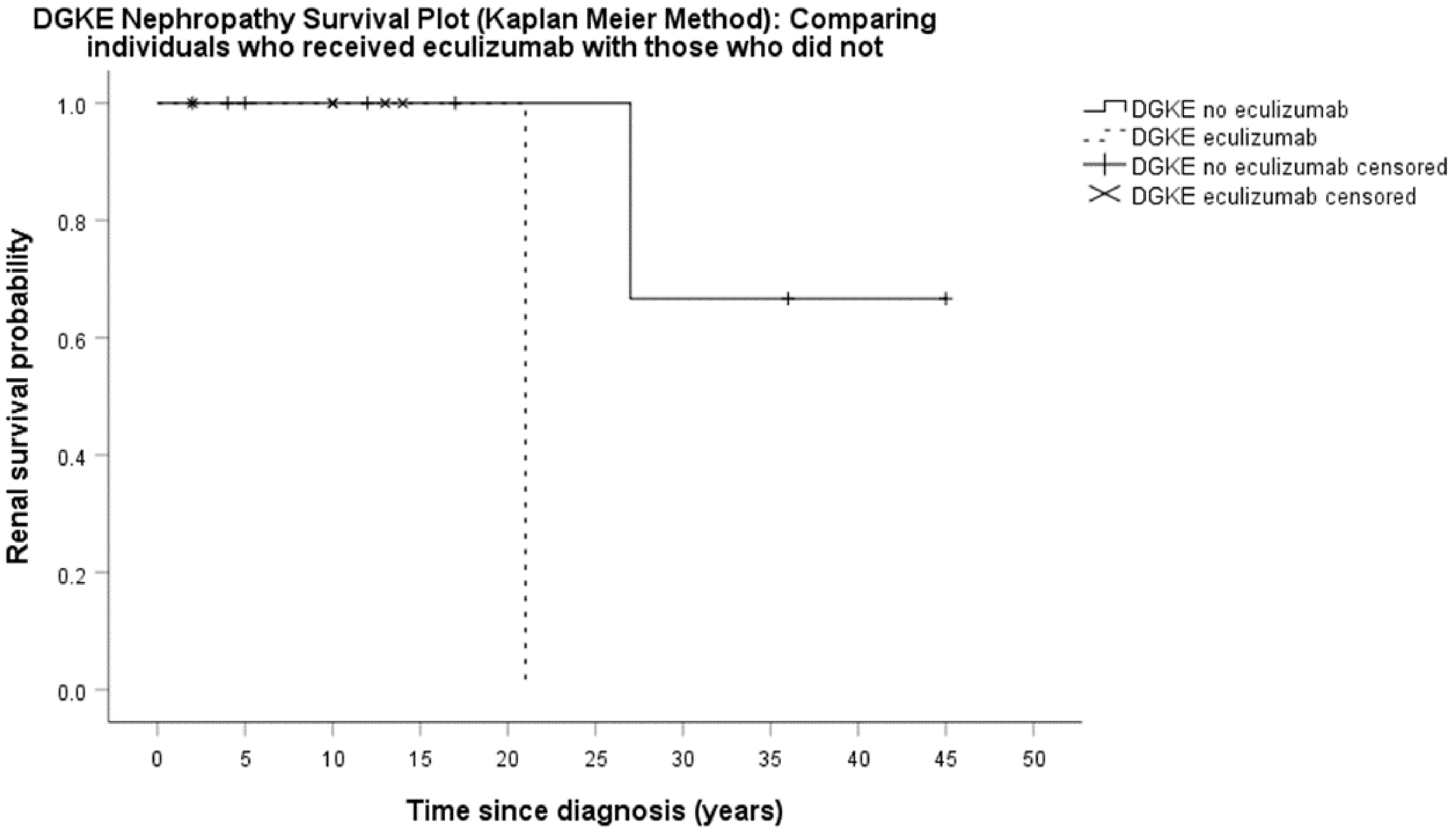

B. Renal and patient survival, including individuals for whom no genetic analysis was possible

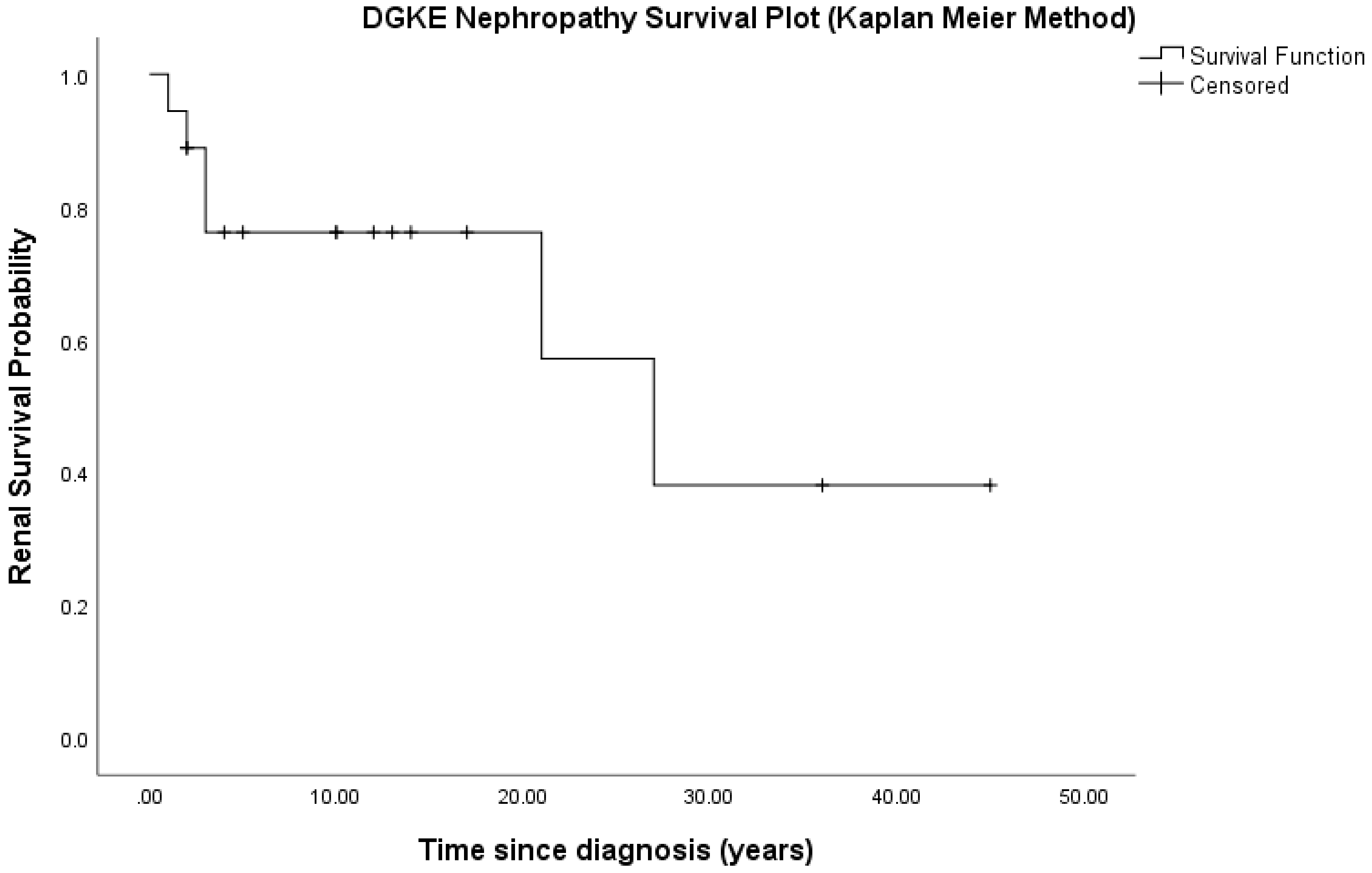

C. Renal and patient survival, including individuals for whom no genetic analysis was possible, comparing individuals who received eculizumab with those who did not

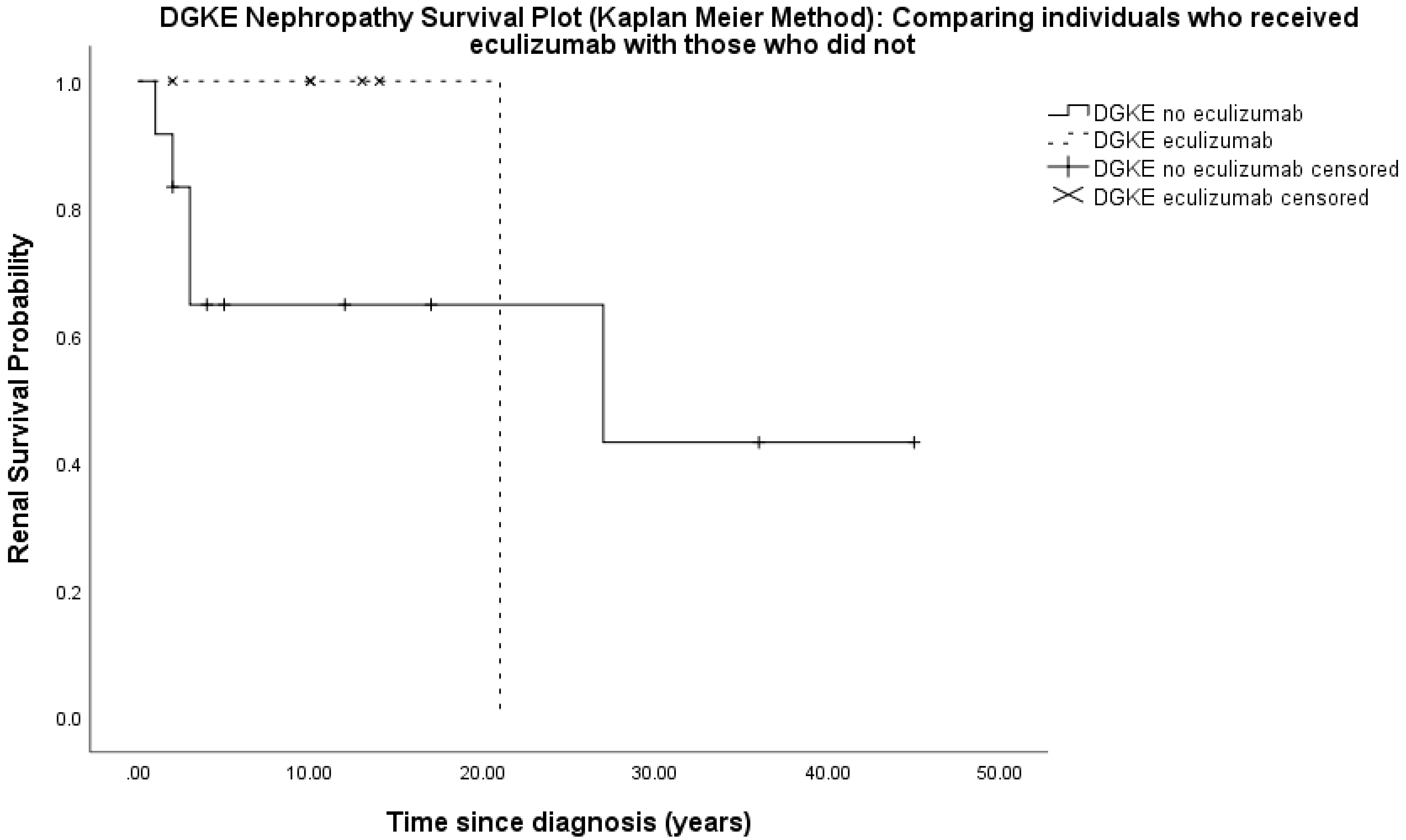

D. Renal survival, incorporating data published by Azukaitis et al., for all published DGKA nephropathy cases

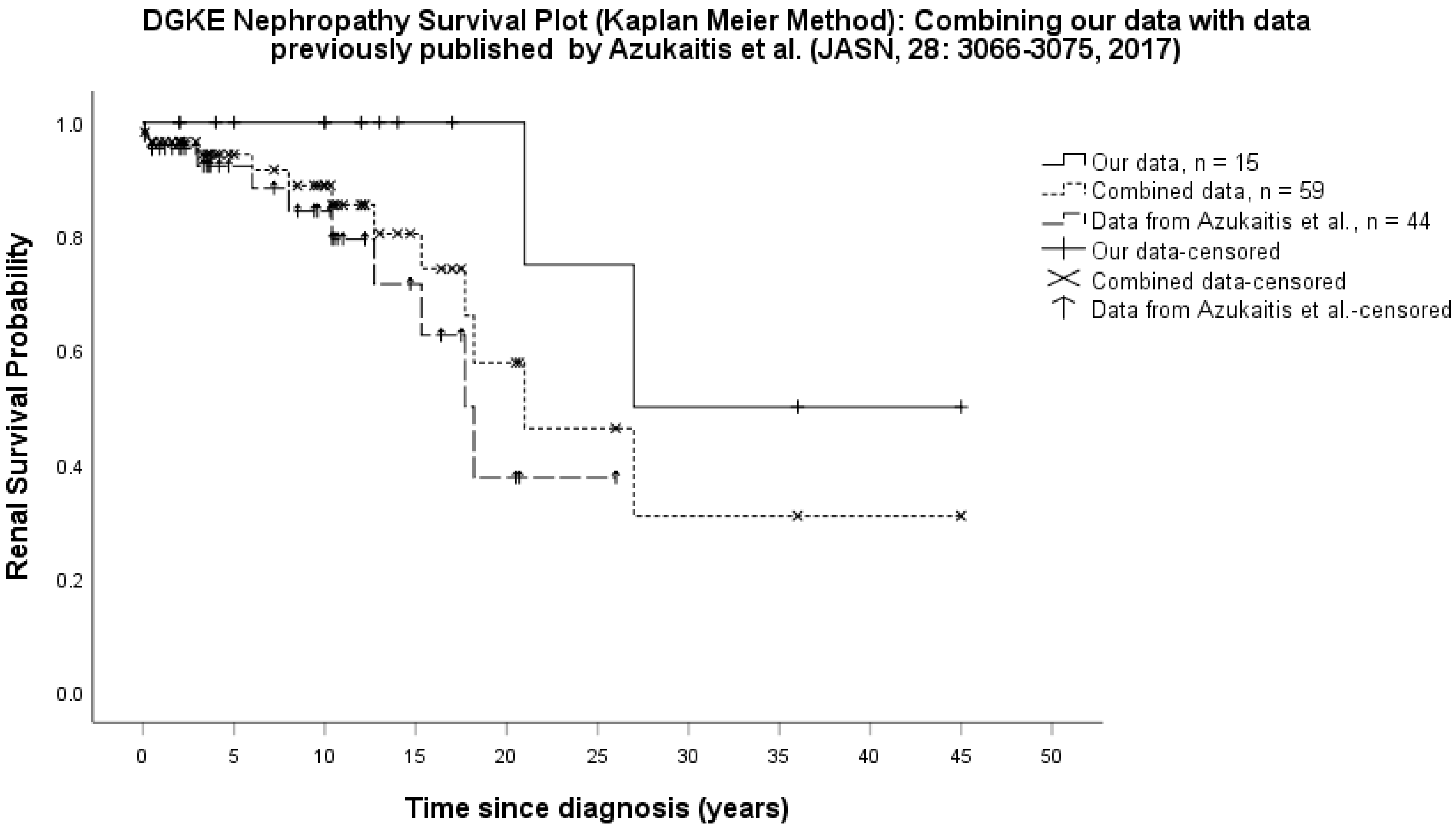

Supplemental Figure 6: Primer design for RNA studies

A. c.1524+2T>C

CTCCAGCTCGCGGCCCCGCGCGCCGGATCGGCGTGCGTGCGGCTGGAGCCTTAAGCGTTTCCCCCGCC
CGGCTTCATCCCTGCTGGCGGCCCAGCGTCGTTCTCCTCCTGCGCGAGGCGGCCAAGGCCTGCTGGCC
CGGAGCCGCGCCTCCACCCGCGCGAGGTATCGTCCTTGGAGAAGATGGAAGCGGAGAGGCGGCCGGCG
CCGGGCTCGCCCTCCGAGGGCCTGTTTGCGGACGGGCACCTGATCTTGTGGACGCTGTGCTCGGTCCT
GCTGCCGGTGTTCATCACCTTCTGGTGTAGCCTCCAGCGGTCGCGCCGGCAGCTGCACCGCAGGGACA
TCTTCCGCAAGAGCAAGCACGGGTGGCGCGACACGGACCTGTTCAGCCAGCCCACCTACTGCTGCGTG
TGCGCGCAGCACATTCTGCAGGGCGCCTTCTGCGACTGCTGCGGGCTCCGCGTGGACGAGGGCTGCCT
CAGGAAGGCCGACAAGCGCTTCCAGTGCAAGGAGATTATGCTCAA**GAATGACACCAAGGTCCTGG**ACG
CCATGCCCCACCACTGGATCCGGGGCAACGTGCCCCTGTGCAGTTACTGTATGGTTTGCAAGCAGCAG
TGTGGCTGTCAACCCAAGCTTTGCGATTACAGGTGCATTTGGTGCCAGAAAACAGTACATGATGAGTG
CATGAAAAATAGTTTAAAGAATGAAAAATGTGATTTTGGAGAATTCAAAAACCTAATCATTCCACCAA
GTTATTTAACATCCATTAATCAGATGCGTAAAGACAAAAAAACAGATTATGAAGTGCTAGCCTCTAAG
CTTGGAAAGCAGTGGACCCCATTAATAATCCTGGCCAACTCTCGTAGTGGAACTAATATGGGAGAAGG
ACTGTTGGGAGAATTTAGGATCTTGTTGAATCCAGTCCAGGTTTTTGATGTAACTAAAACT**CCTCCTA**
TCAAAGCCCTACAACTCTGTACTCTTCTCCCATATTATTCAGCTCGAGTACTTGTTTGTGGAGGGGAT
GGGACTGTAGGGTGGGTCCTGGATGCAGTTGATGACATGAAGATTAAGGGACAAGAAAAGTACATTCC
ACAAGTTGCAGTTTTGCCTCTGGGAACAGGCAACGATCTATCCAATACATTGGGTTGGGGTACAGGTT
ATGCTGGAGAAATTCCAGTTGCGCAGGTTTTGCGAAATGTAATGGAAGCAGATGGAATTAAACTAGAT
CG

exon 1 – 162 bp
exon 2 – 482 bp
exon 3 – 160 bp
exon 4 – 120 bp
exon 5 – 144 bp
exon 6 – 158 bp

predict exon 3 del – 318 bp
WT – 478 bp

DGKE_RNA_Ex2-5f gtagcgcgacggccagtGAATGACACCAAGGTCCTGG
DGKE_RNA_Ex2-5r cagggcgcagcgatgacTGTAGGGCTTTGATAGGAGG

B. c.465-2A>G

GCGGTTTACTTATTCTAT**<u>GGAACCAAAGATTGTTTAGTGC</u>**AAGAATGTAAAGATTTGAATAAAAAAGTTGAGCTA
GAACTGGATGGTGAGCGAGTAGCACTGCCCAGCTTGGAAGGTATTATAGTTCTGAACATCGGATACTGGGGCGGT
GGCTGCAGACTATGGGAAGGGATGGGGGACGAGACTTACCCTCTAGCCAGGCATGACGATGGTCTGCTGGAAGTC
GTTGGAGTATATGGGTCTTTCCACTGTGCTCAGATTCAAGTAAAACTGGCTAATCCTTTTCGAATAGGACAGGCA
CATACAGTGAGGCTGATTTTGAAGTGCTCCATGATGCCAATGCAGGTGGATGGGGAGCCTTGGGCCCAAGGGCCC
TGCACTGTCACCATAA**<u>CTCACAAGACACATGCAATGA</u>**TGTTATATTTCTCTGGAGAACAAACAGATGATGACATC
TCTAGTACTTCGGATCAAGAAGATATAAAGGCGACTGAATAGATGGATGAGGGAGTGAAAACTTTGCATAGAATC
CTCACGCAAGTAGATACATGTTCATCCAAAAGTATTAATAGAAATTCTCTATCAGCTATTCAGTCTTAATTTCAC
TAGTAGTATAATGGGTATACATTTTTGTAAATAGCATCCCCAAACCAGCCAGCCTTCAGTTATTTACAAATGTTT
GTTCTTTTTTCAGCAAAATACTTCAAATGAATAGTATTAACTTACAAAAAGTCACAAAAACTTACATGAGAGTGA
AAATTTGTTATGACTGTTTTGAGAGTGGGACTCACTCTGAAGTATGTGCTGTCTCATGTCTTATTTTTGAACCAT
GCATATGATGGACACACAATGGATGGACACATTATATCTCCAACAAGGTGTGGGTGGAAAGATCAAATTAACCTG
CTTTTTTGAAAGGAAATGATTACTGTCAAACCAGCATGGTTAATTGTGAGCATCCTCC

predicted to lose exon 11 = 112bp or get an extra 4 bases on end of exon 11

Expected product
WT = 428 bp
first 43 bases exon 10 to exon 12 = 215 bp
inclusion of intron 11 = 788 bp

DGKE_RNA_Ex9f gtagcgcgacggccagtGGAACCAAAGATTGTTTAGTGC
DGKE_Ex10_12f gtagcgcgacggccagtTGGATGGTGAGCGAGTAGC
DGKE_Ex10_12r cagggcgcagcgatgacTCATTGCATGTGTCTTGTGAG
